# Temporal and structural genetic variation in reindeer (*Rangifer tarandus*) associated with the pastoral transition in Northwestern Siberia

**DOI:** 10.1002/ece3.6314

**Published:** 2020-04-28

**Authors:** Knut H. Røed, Kjersti S. Kvie, Robert J. Losey, Pavel A. Kosintsev, Anne K. Hufthammer, Mark J. Dwyer, Vasiliy Goncharov, Konstantin B. Klokov, Dmitry V. Arzyutov, Andrei Plekhanov, David G. Anderson

**Affiliations:** ^1^ Norwegian University of Life Sciences Oslo Norway; ^2^ University of Alberta Edmonton AB Canada; ^3^ Ural Branch of the Russian Academy of Sciences Institute of Plant and Animal Ecology Yekaterinburg Russia; ^4^ Department of Natural History The University Museum University of Bergen Bergen Norway; ^5^ Enterprise for Impact (E4i) London UK; ^6^ Scientific Research Institute of Agriculture and Ecology of Arctic Norilsk Russia; ^7^ St. Petersburg University St. Petersburg Russia; ^8^ KTH Royal Institute of Technology Stockholm Sweden; ^9^ The Scientific Center for Arctic Studies Salekhard Russia; ^10^ University of Aberdeen Aberdeen UK

**Keywords:** ancient DNA, Arctic, domestication, mitochondrial DNA, nenets, reindeer husbandry, zooarchaeology

## Abstract

Just as the domestication of livestock is often cited as a key element in the Neolithic transition to settled, the emergence of large‐scaled reindeer husbandry was a fundamental social transformation for the indigenous peoples of Arctic Eurasia. To better understand the history of reindeer domestication, and the genetic processes associated with the pastoral transition in the Eurasian Arctic, we analyzed archaeological and contemporary reindeer samples from Northwestern Siberia. The material represents *Rangifer* genealogies spanning from 15,000 years ago to the 18th century, as well as modern samples from the wild Taĭmyr population and from domestic herds managed by Nenetses. The wild and the domestic population are the largest populations of their kind in Northern Eurasia, and some Nenetses hold their domestic reindeer beside their wild cousins. Our analyses of 197 modern and 223 ancient mitochondrial DNA sequences revealed two genetic clusters, which are interpreted as representing the gene pools of contemporary domestic and past wild reindeer. Among a total of 137 different mitochondrial haplotypes identified in both the modern and archaeological samples, only 21 were detected in the modern domestic gene pool, while 11 of these were absent from the wild gene pool. The significant temporal genetic shift that we associate with the pastoral transition suggests that the emergence and spread of reindeer pastoralism in Northwestern Siberia originated with the translocation and subsequent selective breeding of a special type of animal from outside the region. The distinct and persistent domestic characteristics of the haplotype structure since the 18th century suggests little genetic exchange since then. The absence of the typical domestic clade in modern nearby wild populations suggests that the contemporary Nenets domestic breed feature an ancestry from outside its present main distribution, possibly from further South.

## Graphical abstract

This manuscript demonstrates that the reindeer mitochondrial genome in Northwestern Siberia has gone through massive genetic replacement since medieval times characterized by a significant loss of native mtDNA haplotypes, together with a significant introduction of new haplotypes. The significant temporal genetic shift associated with the pastoral transition suggests that the emergence and spread of reindeer pastoralism in Northwestern Siberia involved the translocation and breeding of a special type of animal with a partly non‐native origin.

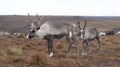

## 1 INTRODUCTION

The domestication of livestock has played a key role in human history. Just as the domestication of sheep, goat, cattle, and pigs were critical elements in the Neolithic transition in many parts of Western Eurasia (Clutton‐Brock, 1999; Zeder, 2008), the domestication of reindeer was important for the development and settlement of the Eurasian Arctic (Bjørklund, 2013; Jernsletten & Klokov, 2002; Kofinas, Osherenko, Klein, & Forbes, 2000). In some parts of this region, people held very small groups of highly trained domestic reindeer to facilitate hunting and fishing since the start of the first millennium (Fedorova, 2000; Gusev, Plekhanov, & Fedorova, 2016). However, what is commonly described as “the pastoral transition” documents a type of large‐scale, extensive reindeer husbandry which is thought to represent one of the most fundamental social transformations to ever take place in the Eurasian Arctic region (Hansen & Olsen, 2014; Krupnik, 1993). This pastoral transition established new relations between humans and animals and led to new settlement and land use patterns across large portions of northern Eurasia. Hence, more knowledge of when, where, and how *Rangifer* pastoralism emerged is key to understand the history of many Arctic communities.

Reindeer (*Rangifer tarandus*) are distributed throughout most of the northern Holarctic region with the domestic form mainly confined to the Eurasian continent. Among the world's total stock of nearly 2,000,000 domestic reindeer, ~2/3 are distributed in Russia and 1/3 in Fennoscandia (Klokov, 2007). Unlike in the Scandinavian countries, where reindeer husbandry is mainly associated with the indigenous Sámi population, a great variety of indigenous peoples across Russia hold domestic reindeer using a wide variety of reindeer herding strategies. Large‐scale nomadic herding, which involves long seasonal migrations and large herds, dominates the Arctic tundra and forest‐tundra areas, while in the more southern taiga and mountain areas reindeer herders typically keep small herds of twenty to a few hundred head that are mainly used to facilitate hunting and transport (Klokov, 2007). Beyond the general distinction of tundra and taiga reindeer husbandry forms, domestic reindeer in Russia subdivided into four official breeds: Nenets, Even, Evenk, and Chukot, which are named after the ethnic groups assumed to have established these breeds with their particular traits and adaptations to their respective environment (Zabrodin & Borozdin, 1989).

Despite decades of research across multiple disciplines, many key issues related to the origins, spread, and intensification of reindeer domestication remain poorly understood. As unambiguous archaeological evidence of domestic reindeer origin and dispersal is unavailable, most theories have instead involved cultural historical models based on surveys of the geographic distributions of different styles of reindeer keeping, its associated equipment, and cultural and linguistic data. Among the early theories, there is the monocentric model of Laufer (1917) who argued that reindeer husbandry originated primarily in the Baĭkal area in southern Siberia at the beginning of our current era (CE [AD]), from where domestic reindeer spread to all other areas. Other scholars suggested polycentric models with more than one origin of initial domestication, implying that reindeer husbandry originated in the Sai͡an and Baĭkal regions of Southern Siberia, as well as in Fennoscandia (Hatt, 1919; Maksimov, 1928; Wiklund, 1918). Recently, the I͡Amal Peninsula (Fedorova, 2000) and the Polar Ural Mountains (Golovnëv, 1993) in Northwestern Siberia have been put forward as two further regions for the independent origin of reindeer husbandry. Many classic models of the diffusion of reindeer husbandry associate it also with the diffusion of domestication gear—halters, tethers, and saddles—from horses to reindeer, and sometimes from horses and dogs to reindeer (Vasilevich & Levin, 1951).

Convincing evidence implies that reindeer have been used by indigenous people for transport, as decoy animals to attract wild reindeer, and in some places for milking, probably long before large‐scale pastoralism emerged. Rock art in Southern Siberia depicting reindeer used for riding and pulling sleds appears to date to the second millennium before CE (Okladnikov & Mazin, 1976). Archaeological remains of “boat‐like coffins”—similar to the Sámi kind of sledge (*gieres*)—have been dated to the same period (Murashkin, Kolpakov, Shumkin, Khartanovich, & Moiseyev, 2016) and may suggest reindeer were used for transport for three millennia in parts of Northern Europe. In Northwestern Siberia, archaeological objects interpreted as reindeer headgear date as early as 100 before CE (Gusev, 2017), which some have argued indicate that reindeer were domesticated and used for transport by that time (Fedorova, 2000; Gusev et al., 2016). However, these fragmentary archaeological findings at best suggest the existence of a small‐scale hunting‐herding tradition, and not the widespread dependence on domestic animals as is associated with the pastoral tradition.

Many scholars assume that reindeer husbandry developed to provide a means of transport to facilitate wild game hunting. Domestic reindeer were probably only killed for sacrifice or when food from game was scarce—as is still the case with many small‐scale forest herding societies (Bjørklund, 2013; Golovnëv, 1993; Ingold, 1988). Over many centuries, this style of reindeer domestication resulted in no significant change in local modes of subsistence, which remained focused on hunting, fishing, and gathering. From the 17th to 18th centuries, the domestic herds of several Arctic people began to grow surprisingly quickly with subsequent emergence of a new form of economy known as pastoralism (Bjørklund, 2013; Ingold, 1988; Krupnik, 1976). The pastoralist transition is characterized by the use of reindeer in nearly all spheres of life starting from transport to a source of skins for clothing and to build dwellings, and as the primary source of food. Besides protecting and controlling their animals, the large herd sizes dictated that these reindeer herders were always on the move and adapted or re‐engineered their earlier semi‐sedentary inventions. As Stépanoff, Marchina, Fossier, and Bureau (2017) have pointed out the scale of the transition inverted the relation between people and reindeer such that the herders had to cater to the needs and the desires of the animals, whom they followed on migrations across the tundra.

The questions related to when, why, and how this pastoral transition took place have been the focus of recurrent scientific debates. In Scandinavia, many authors assume that the collapse in the numbers of wild reindeer from overhunting during 16th to 18th centuries, which accompanied the pressures of colonialism, the market economy, and increased demands for food, led people to change their subsistence strategies (Ingold, 1986; Lundmark, 2007; Vorren, 1973). Others point to long‐term fluctuations in the climate during 17th, 18th, and 19th centuries, producing a favorable cooling cycle that facilitated the explosion of domestic reindeer populations (Krupnik, 1989; Stépanoff et al., 2017). Finally, both Scandinavian and Russian ethno‐historians point to the need for Arctic indigenous groups to keep more animals on hand in order to meet tax and trading obligations with the expanding states around them (Krupnik, 1989; Vorren, 1978). Whatever the cause of the pastoral transition, scholars have speculated whether the rapid growth of domestic reindeer herds was facilitated by the import of a new domestic type (Bjørnstad, Flagstad, Hufthammer, & Røed, 2012; Røed, Bjørklund, & Olsen, 2018), or if wild local stocks were selectively captured and bred to generate a regional domestic type (Golovnëv, 1993).

**FIGURE 1:**
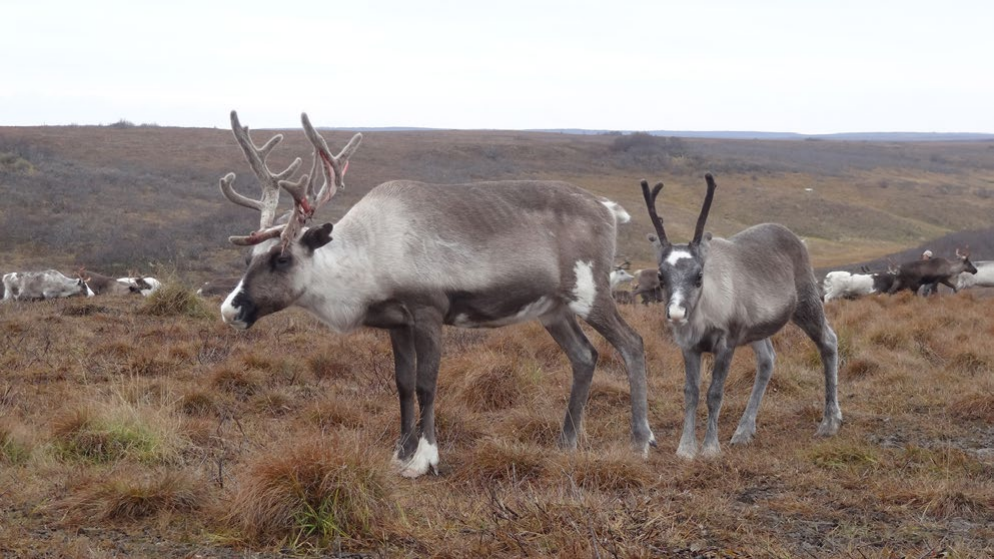
Nenets domestic female reindeer (*Rangifer tarandus*) with calf from Taĭmyr Dolgano‐Nenets District in Northwestern Siberia. Photograph: Vasiliy Goncharov

The domestic reindeer studied are from nomadic herds managed by indigenous Nenetses (Figure 1). Nenetses inhabit the polar regions of Northwestern Siberia and Northeastern Europe and belong to one of several Samoedic linguistic groups whose traditional economy centered on reindeer husbandry (Stammler, 2005). Historical accounts of Samoeds in the 17th century suggest their subsistence centered on hunting and fishing and that their domestic herds were uniformly small scale and used primarily for transport or as decoys to attract wild migratory *Rangifer* (Krupnik, 1976). Beginning in the 18th century, the herd sizes of some families increased greatly, and many families began travel long distances with large domestic reindeer herds depending entirely on them for their dietary and material needs (Krupnik, 1976; Svoboda, Sázelová, Kosintsev, Jankovská, & Holub, 2011). Today Nenetses are the largest indigenous reindeer herding people of the Russian North, altogether herding around 1,000,000 animals (http://www.gks.ru/).

For other livestock species, the use of maternal genetic markers in both extant samples and archaeological remains has proven to be particularly successful in revealing the history of domestication (Almathen et al., 2016; Bollongino et al., 2012; Caliebe, Nebel, Makarewicz, Krawczak, & Krause‐Kyora, 2017; Cieslak et al., 2010; Naderi et al., 2008; Rannamäe et al., 2016). In reindeer, such markers are also greatly useful because they can pinpoint maternal lineages with different origins (Kvie, Heggenes, & Røed, 2016a; Røed et al., 2008; Yannic et al., 2014). The screening of both maternal and nuclear genetic markers of wild and domestic reindeer herds across Eurasia has identified genomic signatures supporting a polycentric hypothesis of the origin of reindeer pastoralism, implying that the Sámi people domesticated their own reindeer independently (Røed et al., 2008). The signatures were particularly evident in the mtDNA haplotype distribution with different haplotype clusters dominating domestic reindeer in Fennoscandia (i.e., **II** and **Ib**) and Northwestern Russia (i.e., **Ie**; Bjørnstad & Røed, 2010; Kvie, Heggenes, & Røed, 2016; Røed et al., 2008). Later, analyses of ancient maternal markers have revealed significant genetic change since medieval times in Northern Fennoscandia reindeer, possibly associated with the emergence of more extensive reindeer husbandry and the import of animals from outside the region (Røed et al., 2018).

In order to address the history of reindeer domestication in Northwestern Siberia, we have analyzed maternal DNA in ancient reindeer bones taken from archaeological sites with genealogies spanning from the Late Pleistocene to the 18th century, and from contemporary wild and domestic reindeer. Specifically, we tested for a temporal genetic change in reindeer associated with the pastoral transition in the region, determined when this change occurred, and whether the local wild stocks were used as basis to form the contemporary domestic breed.

## 2 METHODS

### 2.1 Archaeological material

**FIGURE 2:**
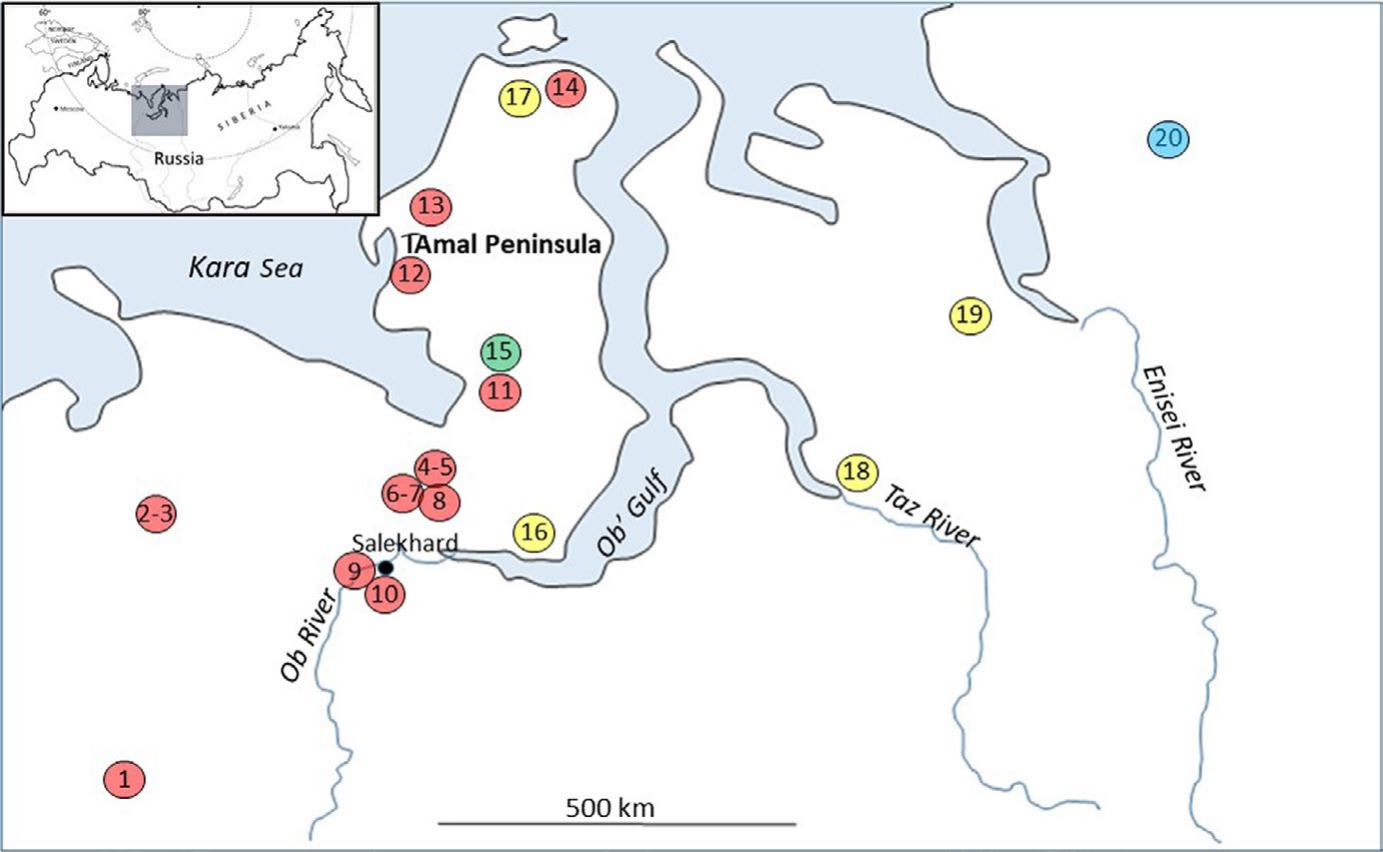
Locations of sampling sites with sample codes of archaic (sample codes 1–14 in red and sample code 15 in green) and contemporary domestic (yellow, sample codes 16–19) and wild reindeer (blue, sample code 20) in Northwestern Siberia. See Table 1 and Appendix S1 for more information of samples and localities

Altogether 299 samples from bones, teeth, and antlers were obtained from archaeological faunal assemblages at twelve excavated sites in the I͡Amal‐Nenets Autonomous District (I͡ANAD), the Nenets Autonomous District (NAD), and the eastern portion of the Komi Republic (KR), with ages spanning from ~15,000 years before present (BP) to ~300 BP (Table 1; Figure 2). The sites were caves or rock shelters without any certain signs of human presence (sample codes 1–8), a ritual site (sample code 9), or human settlement sites (sample codes 10–15). Hunting and fishing economies in tundra environments characterize the five archaeological sites located on the I͡Amal Peninsula (sample codes 11–15). Here, the many sandy riverbanks have created dwelling spaces for mobile hunters for thousands of years, several with large assemblages of animal remains dominated by *Rangifer*. Artifacts found at some sites suggest the presence of domestic reindeer (for more information and references to each site see Appendix S1). Site 15 is the youngest site, and dates to circa AD 1700, and therefore is marked separately in Figure 2. The site and bone assemblage chronology are based on the calibrated radiocarbon dating of 39 reindeer samples used in this study (Figure S1 in Appendix S1) together with radiocarbon dating of samples from the artifact‐bearing strata prior to this study (Hufthammer, Svendsen, & Pavlov, 2018; Losey et al., 2018; Hufthammer, Henriksen, and Pavlov, pers. com.). Samples from sites with archaeological stratification, and where radiocarbon dating supported stability, were separated into upper and lower excavation layers (Table 1; Appendix S1). Suitable reindeer teeth, bones, or antlers were carefully selected, based on osteological determinations. When possible, different individuals were identified and classified according to morphology, size, and age group, to ensure that the samples within each site were from different animals.

**TABLE 1:** Amount of genetic variation in CR in archaic reindeer (sample codes 1–15), domestic reindeer (sample codes 16–19) and wild reindeer (sample code 20) in Northwestern Siberia. The archaic material at Pymva‐Shor and I͡Anganapė‐2 and 3 are separated in lower and upper layer (LL and UL) due to age stratification of the material. Age is given as approximate calibrated years before present (BP), *N* = number of individuals, *N*_h_ = number of different haplotypes. Haplotype and nucleotide diversity are given with ± *SD* in brackets

Sample type	Sample code	Location/layer	Institute journal code	Location/site type	Age (BP)	*N*	*N*_h_	Haplotype diversity	Nucleotide diversity	Mean haplotype pairwise difference
Ancient	1	Podcherem	J.S. 1161	Cave/rock shelter	15,000	19	14	0.953 (0.036)	0.032 (0.018)	6.006 (2.995)
Ancient	2	Pymva‐Shor (LL)	J.S. 950	Cave/rock shelter	13,000	2	2	1.000 (0.500)	0.026 (0.028)	5.000 (3.873)
Ancient	3	Pymva‐Shor (UL)	J.S. 950	Cave/rock shelter	5,900	14	14	1.000 (0.027)	0.030 (0.017)	6.603 (2.768)
Ancient	4	I͡Anganapė‐2 (LL)	S. 976	Cave/rock shelter	3,500	6	6	1.000 (0.096)	0.033 (0.021)	6.333 (3.500)
Ancient	5	I͡Anganapė‐2 (UL)	S. 976	Cave/rock shelter	1,800	23	15	0.960 (0.022)	0.032 (0.018)	6.126 (3.024)
Ancient	6	I͡Anganapė‐3 (LL)	S. 1001	Cave/rock shelter	2,700	21	15	0.962 (0.026)	0.032 (0.018)	6.067 (3.009)
Ancient	7	I͡Anganapė‐3 (UL)	S. 1001	Cave/rock shelter	900	19	14	0.959 (0.031)	0.030 (0.017)	5.743 (2.877)
Ancient	8	I͡Anganapė‐4	S. 1002	Cave/rock shelter	700	3	3	1.000 (0.272)	0.032 (0.026)	6.000 (3.928)
Ancient	9	Ust’‐Poluĭ	S. 736	Forest/tundra ritual site	1,700	1				
Ancient	10	Zelenai͡a Gorka	S. 852	Forest/tundra settlement	650	23	16	0.949 (0.033)	0.037 (0.020)	7.115 (3.464)
Ancient	11	I͡Arte‐6	S. 677	Tundra settlement	900	41	25	0.983 (0.017)	0.037 (0.020)	7.028 (3.369)
Ancient	12	I͡Uneta‐i͡akha‐14	S. 2442	Tundra settlement	700	15	12	0.962 (0.040)	0.038 (0.021)	7.124 (3.541)
Ancient	13	Tiuteĭ‐Sale‐1	S. 783	Tundra settlement	800	15	12	0.920 (0.040)	0.034 (0.019)	6.476 (3.247)
Ancient	14	Khėkhė‐i͡akha‐1	S. 2393	Tundra settlement	700	8	6	0.929 (0.084)	0.034 (0.021)	6.464 (3.423)
Ancient	15	Khali͡ato‐1	S. 632	Tundra ritual site	300	13	4	0.756 (0.070)	0.016 (0.010)	3.077 (1.709)
				Ancient total		223	111	0.986 (0.002)	0.034 (0.018)	6.532 (3.100)
Extant	16	I͡Amal South		Domestic herd	Extant	56	11	0.639 (0.064	0.014 (0.008)	2.608 (1.416)
Extant	17	I͡Amal North		Domestic herd	Extant	23	7	0.704 (0.089)	0.015 (0.009)	2.806 (1.537)
Extant	18	Taz‐Nenets		Domestic herd	Extant	13	8	0.859 (0.089)	0.022 (0.013)	4.154 (2.208)
Extant	19	Eniseĭ‐Nenets		Domestic herd	Extant	45	12	0.770 (0.047)	0.016 (0.009)	2.986 (1.590)
				Domestic total	Extant	137	21	0.733 (0.030)	0.016 (0.009)	3.031 (1.591)
Extant	20	Taĭmyr		Wild population	Extant	60	29	0.959 (0.011)	0.030 (0.013)	5.645 (2.746)

### 2.2 Contemporary reindeer

DNA from 173 skin, muscle, and blood samples from contemporary domestic and wild reindeer were extracted and analyzed. The four domestic sample sets were from separate and widely spread districts in I͡ANAD and the western part of the Taĭmyr Dolgano‐Nenets District (TDND; Figure 2). The sample named I͡Amal South (sample code 16) consists of pooled samples from different brigades within the neighboring territories of the I͡Ar‐sale *sovkhoz* and Panaevsk *sovkhoz* in the southern I͡Amal Peninsula. Most of these brigades perform long migrations between the winter pastures south to the Ob' Gulf toward the summer pastures along Kara Sea coast. The sample set I͡Amal North (sample code 17) is from reindeer close to Malygina Strait at the north end of the I͡Amal Peninsula. Their seasonal migration pattern is mainly within the northern territory of I͡Amal *sovkhoz*. The Taz‐Nenets samples (sample code 18) were from a herd in the Taz District located directly east of the I͡ANAD on the opposite side of the Ob' Gulf from the I͡Amal Peninsula. These eastern Nenets families move their herds annually from the town of Tazovsk on the Taz River all the way to the western part of the TDND. The Eniseĭ‐Nenets samples (sample code 19) were from the reindeer herding families who travel around the settlements of Tukhard and Nosok located at the western part of TDND. Most of the ~100,000 Nenets reindeer in TDND are situated in this area.

The wild reindeer samples were from the Taĭmyr population inhabiting the Taĭmyr Peninsula and adjoining territories including southern and western parts of TDND. In addition to large population size, with numbers fluctuating during the last 50 years from several hundred thousand to a million individuals, the absence of barriers that could promote its isolation, characterize this population (Kolpashchikov, 2000). The contemporary wild reindeer sample set (sample code 20) used in this study consists of 36 samples obtained from the western or central Taĭmyr Peninsula together with 24 western or central Taĭmyr reindeer sequences downloaded from the GenBank (KX094725–KX094748, Table S3 in Appendix S1).

### 2.3 Laboratory methods

DNA isolation and amplification setup of the archaeological material were undertaken in spatially separated lab facilities with standard precautions for working with ancient samples (Hofreiter, Serre, Poinar, Kuch, & Paabo, 2001; Wandeler, Hoeck, & Keller, 2007). Each sample was cleaned, and the outer surface removed before being drilled to obtain approximately 10–20 mg of powder. DNA was extracted using DNeasy^TM^ Tissue Kit (Qiagen) following the protocol described in Bjørnstad and Røed (2010). All equipment and working surfaces were cleaned using sodium hypochlorite, ethanol, or UV light. A 266 base pair (bp) long fragment of the mitochondrial control region (CR) was analyzed with primer sequences, PCR amplification, and sequencing as given in Røed et al. (2014). To test for contamination and DNA degradation, blank extraction and controls were used in each amplification and only DNA sequences that could be replicated from at least two independent amplifications were accepted. The degree of nucleotide misincorporation in the amplified products was analyzed in 20 accepted samples distributed among different sampling sites (Table S2 in Appendix S1) by a third PCR amplification with subsequent cloning in plasmid vector by using the TOPO TA cloning kit (Invitrogen). We compared clones from each individual PCR product and identified misincorporations by looking for substitutions in each of the cloned sequences, compared with the consensus sequence of the amplification.

DNA was extracted from contemporary blood, skin, and tissue samples using DNeasy Blood and Tissue Kit (Qiagen) following the manufacturer's protocol. A 503‐bp long fragment of mitochondrial CR was amplified and sequenced as given in Kvie, Heggenes, and Røed (2016). All sequences are deposited in the Sequence Database at the National Centre for Biotechnical Information (NCBI; GenBank ID: MT146049–MT146444, Table S3 in Appendix S1).

### 2.4 Data analyses

To obtain the maximum number of sequences for comparison, the amplicons were trimmed to 190 and 374 bp for the ancient and contemporary CR alignments, respectively. In this study, we compared the same 190 bp alignment in modern and ancient material. arlequin v3.5 (Excoffier & Lischer, 2010) was used to estimate nucleotide and haplotype diversity and to obtain site pairwise *F*_ST_ estimates based on haplotype frequencies. The statistical significance was evaluated using 1,000 permutations. Average numbers of nucleotide differences were calculated in DnaSP v5 (Librado & Rozas, 2009). The program SAMOVA v1.0 (Dupanloup, Schneider, & Excoffier, 2002) was used to define groups of sample sets that are spatially and temporally homogeneous and maximally genetically differentiated from each other. Differences in average nucleotide diversity measures between sample sets were assessed by analyses of variance (ANOVA) and Wald statistics using the lm‐function in R (R Core Team, 2019). Visual expectation of standard diagnostics tools revealed no clear deviations from the underlying assumptions for linear models.

BEAST v1.8.0 (Drummond, Suchard, Xie, & Rambaut, 2012) was used to construct a Bayesian phylogeny of the identified haplotypes. The analysis was run with an HKY G + I substitution model with strict molecular clock for 10^9^ generations with trees sampled every 10^5^ iterations. TreeAnnotater v.1.7.5 (Drummond et al., 2012) was used to create a maximum clade credibility tree that represents the posterior distribution. Sequences from previously described CR haplotype clusters were included in a separate Bayesian analysis to designate sequences from the current study to previously described haplotype clusters (Kvie, Heggenes, & Røed, 2016). Convergence for the phylogeny was assessed in TRACER (Rambaut, Xie, & Drummond, 2014) giving the effective sample size for all parameters above the general recommendation (ESS > 200). Genealogical relationships among haplotypes were examined by constructing a median‐joining network using Network v4.6 (ref.fluxus‐engineering.com).

## 3 RESULTS

Of the 299 ancient samples, 223 produced reproducible mtDNA sequences (Table S3 in Appendix S1). All 20 PCR products amplified for cloning gave identical sequences to these consensus sequences. After cloning, seventeen cloned products gave a predominance of clones identical to the consensus sequence, and two products gave respectively two and one clone identical, while one product displayed all twelve clones analyzed to be different, none of which were identical to the consensus sequence. We analyzed altogether 199 clone sequences and identified a total of 243 nucleotide misincorporations mainly represented by C/G to T/A transitions (89%) together with some T/A to C/G transitions (9%) and few transversions (2%; Table S2 in Appendix S1). The dominate transitions are misincorporations typically seen when sequencing ancient DNA (Stiller et al., 2006). In all, both the repeated PCR and the dominance of clones identical to the consensus sequences give evidence of the consensus sequences to be little affected by the misincorporations and are thus considered to be mainly reliable.

Standard estimates of DNA polymorphism of the 190 bp archaeological sequences showed a high degree of variation with 111 haplotypes, and haplotype and nucleotide diversity equal to 0.986 and 0.034, respectively (Table 1). From the 197 contemporary wild and domestic reindeer, the 374 bp CR fragment defined 53 haplotypes, 23 in domestic and 33 in wild reindeer. After adjusting the sequences to the same 190 bp as for the ancient material, the nucleotide substitution of the extant material defined 47 haplotypes, with haplotype and nucleotide diversity equal to 0.859 and 0.024. The 190 bp fragment defined 21 and 29 haplotypes in, respectively, contemporary domestic and wild reindeer, with haplotype and nucleotide diversity equal to 0.733 and 0.016 for the domestic and 0.959 and 0.030 for the wild reindeer (Table 1). The pooled ancient and extant 190 bp alignment defined altogether 137 different haplotypes among which 82 were singletons.

**FIGURE 3:**
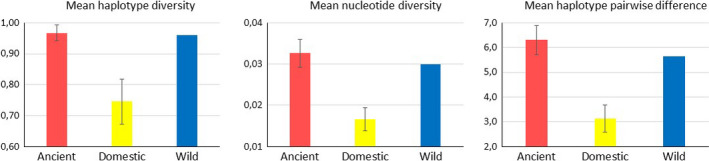
Mean control region diversity and haplotype pairwise difference estimates, with ±1 *SD*, in reindeer across sample codes 1–14 (ancient), sample codes 15–19 (recent domestic) and sample code 20 (contemporary wild) in Northwestern Siberia

Pairwise genetic differences among sample sites (Table 2) gives a clear pattern of low or no genetic differentiation among most of the archaeological sites (sample codes 1–14) with the exception of the youngest archaeological site (Khali͡ato‐1, sample code 15). Similarly, low or no genetic differentiation characterized the four sample sets of modern domestic reindeer (sample codes 16–19) as well as the archaeological Khali͡ato‐1 site. The contemporary wild reindeer (sample code 20) showed high and significant differentiation from modern domestic reindeer and the Khali͡ato‐1 site, but little differentiation from most of the other archaeological sites. This result was supported by the SAMOVA analysis indicating that the data is best divided into two groups (*K* = 2) of sample sets, that is, codes 1–14 together with 20 versus codes 15–19 on the basis of the among‐group partitioning of molecular variance (Figure S1 in Appendix S1). The low mean FST value among sample codes for these two sets, that is, 0.028 ± 0.023 and 0.038 ± 0.042, respectively, gives evidence of low genetic differentiation within these two sample sets. On the contrary, the high value for the mean pairwise differences observed between the two sample sets (0.264 ± 0.092) indicates a clear genetic separation and the occurrence of a distinct temporal genetic shift associated with the domestic reindeer. This strong division between the data sets comprising the most ancient samples in addition to contemporary wild reindeer, and a more recent domestic set, is associated with changes in the estimates of genetic variation as illustrated by the 111 haplotypes observed among 223 ancient specimens compared to 21 haplotypes in 137 domestic specimens (Table 1). The ANOVA revealed a significant reduction in both average haplotype diversity (*F* = 74.43, *p* < .01, *R* ^2^ = .83), nucleotide diversity (*F* = 115.15, *p* < .01, *R* ^2^ = .89) and nucleotide pairwise differences (*F* = 132.18, *p* < .01, *R* ^2^ = .90) in the recent domestic sample set, compared to the wild sample set (Figure 3). Notably, as many as 11 of the 21 haplotypes present in the modern domestic reindeer were not present among the archaeological material nor the contemporary wild reindeer.

**TABLE 2:** Pairwise genetic differences (*F*_ST_) between reindeer obtained at various archaeological sites (sample codes 1–15) and sets of contemporary reindeer (sample codes 16–20) in Northwestern Siberia. The sample locations are as given in Table 1 and Figure 2. Sample codes with less than four samples are not included in the analyses. Asterisks give differences at significant levels <0.01

Sample code	1	3	4	5	6	7	10	11	12	13	14	15	16	17	18	19
1																
3	0.000															
4	0.025	0.005														
5	0.049	0.013	0.029													
6	0.060*	0.028	0.000	0.024												
7	0.043	0.042	0.027	0.024	0.000											
10	0.057*	0.027	0.030	0.059*	0.023	0.038										
11	0.039	0.014	0.016	0.003	0.000	0.000	0.019									
12	0.081	0.042	0.000	0.036	0.005	0.062	0.042	0.010								
13	0.040	0.019	0.097	0.003	0.066	0.023	0.038	0.008	0.074							
14	0.039	0.025	0.000	0.046	0.000	0.006	0.002	0.000	0.000	0.070						
15	0.232*	0.128*	0.283*	0.165*	0.246*	0.266*	0.200*	0.170*	0.175*	0.305*	0.206*					
16	0.374*	0.305*	0.453*	0.297*	0.416*	0.420*	0.385*	0.308*	0.308*	0.495*	0.399*	0.098*				
17	0.311*	0.229*	0.385*	0.246*	0.349*	0.361*	0.305*	0.250*	0.256*	0.411*	0.322*	0.027	0.000			
18	0.189*	0.101	0.231*	0.125*	0.222*	0.227*	0.179*	0.148*	0.113*	0.270*	0.179*	0.000	0.026	0.000		
19	0.268 *	0.186*	0.355*	0.160*	0.281*	0.281*	0.284*	0.193*	0.167*	0.385*	0.263*	0.046	0.094*	0.091	0.000	
20	0.059*	0.003	0.045	0.020	0.016	0.025	0.035	0.004	0.043	0.018	0.031	0.150*	0.314*	0.256*	0.153*	0.187*

**FIGURE 4:**
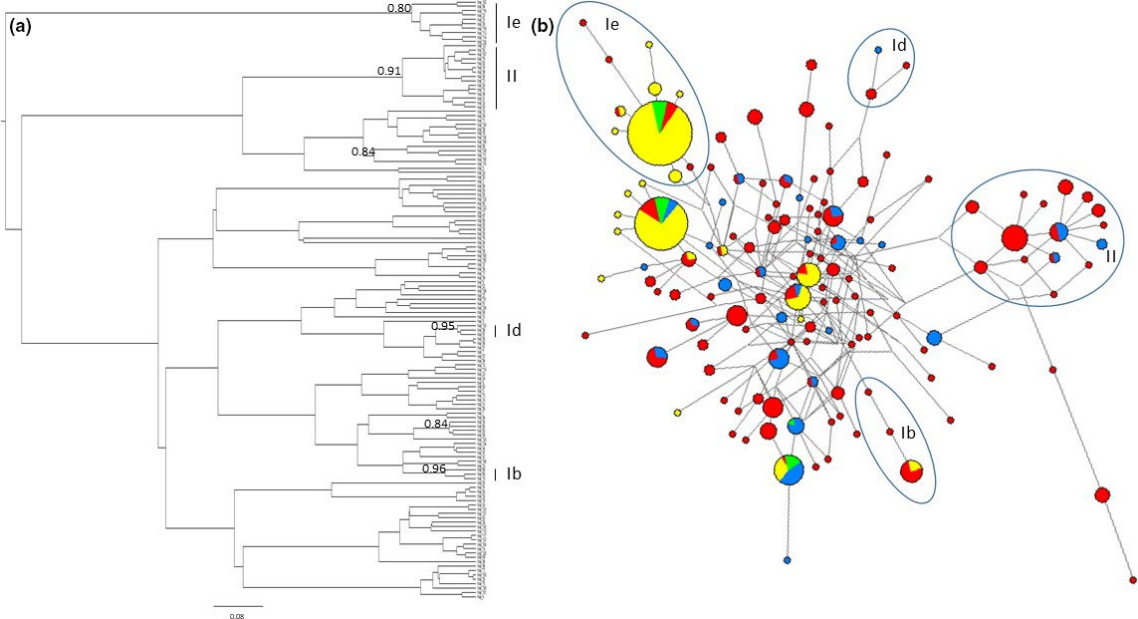
Bayesian consensus tree (a) and network (b) for ancient and contemporary reindeer in Northwestern Siberia inferred using 190 bp of the control region. Bayesian posterior probabilities >.80 provided at the tree nodes. Clade labels used throughout the text are indicated as vertical bars beside the tree and as encircled haplotypes in the network. Each colored sphere represents unique haplotypes with the area proportional to the number of reindeer sharing a haplotype, and with colors representing the sample codes 1–14 (archaic) in red, sample code 15 (archaic) in green, sample codes 16–19 (modern domestic) in yellow and sample code 20 (contemporary wild) in blue

The Bayesian phylogenetic tree reconstruction of the 137 haplotypes identified several medium to well‐supported clusters/subclusters (Figure 4a). Some of these were recognized as the same clusters previously reported to dominate modern domestic reindeer in Fennoscandia (labeled **II**, **Ib)** and in Northwestern Russia (labeled **Ie;** Røed et al., 2008; Bjørnstad & Røed, 2010; Kvie, Heggenes, & Røed, 2016). The MJ network (Figure 4b) revealed cluster **II** to be present only among ancient and modern wild reindeer, while modern domestic reindeer together with the youngest archaeological Khali͡ato‐1 site clearly dominated subcluster **Ie**. Subcluster **Ie,** comprising ten haplotypes in 86 samples, had a star‐like haplotype pattern with one haplotype at high frequency (0.83) and with all other haplotypes radiating from this by one to three mutations (Figure 4b). Such a star‐like pattern is thought to have been produced by an ancient population expansion with the most common haplotype assumed to be the ancestral haplotype (Slatkin & Hudson, 1991). This ancestral haplotype dominated all modern domestic herds (frequency range 0.27–0.57) and the youngest archaeological site code 15 (frequency 0.38), but was absent in contemporary wild reindeer and present in only four of the older sites (one in each of sample code 3, 5, 10 and 13). Cluster **II** was represented by 15 haplotypes in 43 samples, of which all were found in contemporary wild reindeer (*n* = 6) or in the older sample codes 3–13 (*n* = 37). Subcluster **Ib** contained three haplotypes in 11 samples, two in modern domestic reindeer and nine in ancient reindeer (sample codes 3–12).

## 4 DISCUSSION

Our analysis of the mitochondrial CR in contemporary and archaeological reindeer in the vast Nenets‐occupied region reveals two distinct genetic clusters. The first corresponds to the modern wild reindeer together with the older sample sets ranging from the late Pleistocene to the late medieval periods covering large areas from I͡Amal in the East to the Ural Mountains in the West. The second includes those from widely separated contemporary domestic herds from I͡ANAD and the western part of the TDND together with the relatively recent archaeological site dated to 300 BP. The high proportion of temporal sample overlaps on I͡Amal and the fact that nearly all archaeological sites clustered together with contemporary wild reindeer, and all modern domestic herds assigned to the other cluster, gives strong evidence that these highly differentiated clusters represent the maternal gene pools of wild and modern domestic reindeer respectively. Based on these results, the mitochondrial CR appears to be an appropriate marker to study genetic processes associated to the pastoral transition in Northwestern Siberia.

### 4.1 The pastoral transition and the genetic shift

The high levels of genetic variation within a homogeneous genetic structure found within the wild gene pool both temporally and spatially suggest that relatively large populations of wild reindeer occupied the region since late Pleistocene. The significant genetic shift associated with the late‐dated archaeological site Khali͡ato‐1 is striking and points toward a fundamental genetic change on the I͡Amal that seems to have been completed by the start of the 18th century. This change coincides very roughly with the period associated with the onset of the pastoral tradition. Using historical sources, Krupnik (1989) associates the practice of holding of large herds of domestic reindeer to the beginning of the 18th century, proceeding to a “transition” to pastoralism by the middle of the 19th century, and the establishment a fully socio‐economic “revolution” by the end of the 19th century. Although we only have evidence from one archaeological site dating to this period, it seems reasonable to link the genetic shift to this pastoralist transition. According to our data, the genetic shift on I͡Amal could have happened anywhere between the 15th and 18th centuries. With a greater variety of samples, it might be possible to date this genetic shift more precisely.

It should be noted that others have interpreted the many artifacts found at sites on the I͡Amal Peninsula as signs of a social complexity and a remarkable inter‐regional standardization of techniques built on large‐scale reindeer transport and large‐scale reindeer husbandry, beginning as early as 2,000 BP (Gusev et al., 2016). If this was the case, according to our data, these processes must have involved domesticated animals that were genetically similar to the wild stock stretching back to the late Pleistocene.

The typical wild gene pool pattern which is dominant in all archaeological sites prior to the 15th century, suggests that people in the region relied primarily upon wild reindeer for food. However, it is possible that these hunting groups also held a small number of domestic reindeer to use as hunting decoys or for transport. The four samples presenting a typically modern domestic genetic signature (most common cluster **Ie** haplotype) at sites 3, 5, 10 and 13 could indicate that a small number of animals with a genetic constitution similar to those of the domestic reindeer today were present on I͡Amal since as far back as 6,000 BP. If local wild reindeer were tamed (cf. Golovnëv, 1993), we would not be able to genetically distinguish them from the wild reindeer. If a different single animal was imported for a special purpose, for example to serve as decoy reindeer, it is likely that its signature would be rare. Likely these exotic animals were held in small numbers, were highly valued, and were not slaughtered for food (except in times of great starvation; Krupnik, 1993). Such sparse remains would naturally be lost or diluted among the larger amounts of bone material deposited from the remains of wild migratory reindeer. It is possible that the remains of domestic reindeer may have been curated differently to those of wild reindeer. We know that contemporary hunter‐fisher Nenetses create offerings of domestic reindeer bones at sacred sites on the surface (Haakanson, 2000). Further, at the recent site Khali͡ato‐1, *Rangifer* remains were deposited on the surface as a ritual sculpture (Kardash & Sokolkov, 2016, cf. Appendix S1). Surface deposition would be poorly preserved across the millennium. What can be concluded is that the animals presenting cluster **Ie** features appeared first in a scattering of archaeological sites in small numbers, and then came to rapidly dominate the signatures of the domestic reindeer population of this region.

The contemporary domestic population also presents a limited number of haplotypes with reduced mean pairwise haplotype differences in the maternal gene pool. It also displays several new haplotypes not present in the old and modern wild gene pool. This suggests that the Nenets pastoral transition found its origins on a limited number of individuals with a maternal ancestry comprising partly of non‐native origin. It is possible that the rapid growth in herd sizes that has been documented from the 17th‐19th centuries facilitated the development of a unique type based on small numbers of imported pioneers. Our data therefore associates the emergence of pastoralism with the actual translocation of a special type of animal, as has been also reported for pastoral transition in Fennoscandia (Røed et al., 2018). This strategy of importing specialized domestic stock is also seen for several other domesticated animal species (Clutton‐Brock, 1999; Larson & Burger, 2013).

The pattern of temporal genetic change for the Nenets reindeer is very similar to what has been reported for reindeer in northern Fennoscandia, where reindeer appeared to have gone through a massive maternal mtDNA replacement during the 16th and 17th centuries, suggested to be associated to the onset of pastoralism (Bjørnstad et al., 2012; Røed et al., 2018). Here, the collapse in the numbers of wild reindeer, together with pressures from colonialism and market economy led people to keep more animals at hand (Ingold, 1988; Lundmark, 2007; Vorren, 1973). It is interesting to note that other reindeer herding peoples, such as Chukchis and Koriaks much further east in Russia also experienced a rapid growth in the size of their domestic herds in the 17th and 18th centuries (Krupnik, 1993).

The near‐simultaneous emergence of pastoralism across the Eurasian Arctic which generally accompanied the decrease of the local wild reindeer (Ingold, 1986; Klein, 1980; Lundmark, 2007; Syroechkoveskii, 1995) suggests that there might have been some general drivers. This could be the onset of the Little Ice Age when most of the Arctic experienced the coldest sustained temperatures of the past 800 years, with the coldest interval occurring between the 17th and mid‐19th centuries (Briffa et al., 2013; Kaufman et al., 2009). *Rangifer* respond well to damp cool summers and deeply cold winters (Weladji, Klein, Holand, & Mysterud, 2002). Increasing domestic herd sizes perhaps allowed for increased human mobility that in turn greatly facilitated the hunting of wild reindeer (Krupnik, 1993; Stépanoff, 2017). Consequently, at the turn of 19th century, the decline of the wild reindeer population did not precede but rather followed the increase in domestic herds. According to this scenario, the main facilitating factor of the transition from hunting to pastoralism was climate, although local political and economic factors may have been influential as they incited herders to keep large herds. Following the initial transition to pastoralism when people shifted toward subsisting primarily on domestic animals, further reduction and eventually depletion of the wild reindeer may have taken place due to the challenges of the coexistence of large wild and domestic herds (Baskin, 2005; Klein, 1980).

### 4.2 The genetic structure and ancestry of I͡Amal‐Nenets domestic reindeer

The contemporary domestic breed displays several CR haplotypes not present in the wild gene pool. Admittedly, the data show a large number of haplotype singletons implying that more haplotypes might be discovered with a greater variety of archaeological cases. Most private domestic haplotypes were closely related to a few common haplotypes suggesting that the domestic breed was built from a few main maternal lineages. The fact that domestic herds exploded in size over the last few centuries enhance the probability that some of these unique private haplotypes represent mutations in the hypervariable part of the CR over this time span. However, the high proportion of private domestic haplotypes (i.e., about 50%), and the small fraction haplotypes belonging to the domestic lineages among the archaeologic sites 1–14, nevertheless suggest that the dominant domestic type of today had its origins in animals imported from other areas.

Several mechanisms could have been involved in introducing new maternal genomes to the region. This polar region experiences periodic crashes in the number of reindeer due to poor weather or spring icing events (Briffa et al., 2013; Forbes et al., 2016; Klokov, 2011). Both historical sources and oral history document significant collapses of reindeer due to diseases or icing events in 1911 and 1922 (Podkorytov, 1995). It is therefore conceivable that subsequent to previous population crashes the herds were replenished from other reindeer‐herding regions to the south or to the east. There are also documented state‐led experiments in translation during the Soviet‐era of importing domestic reindeer from Chukotka to improve local domestic herds (Podkorytov, 1995). Furthermore, low genetic differentiation characterizes the domestic reindeer herds analyzed in this study. Both the long‐term pattern of reindeer transfer due to dowry and friendship exchanges and later state‐led breeding and exchange of particular animals may have contributed to the development of a distinct and relatively homogeneous genetic structure for the Nenets domestic reindeer breed seen today.

If we reflect on the fact that the same homogeneous genetic signature is present from as late as the 18th century site on I͡Amal, it would seem that the genetic shift must have predated the era of Soviet zootechnics. Further, the fact that the mean haplotype differences are low among the domestic reindeer point toward a common evolutionary history characterizing the domestic maternal gene pool. Most private haplotypes in the domestic population were within the CR cluster with the signature of ancient population expansion (**Ie**, Figure 4b). This, together with the dominance of domestic animals among also the ancestral haplotype of this cluster, may indicate a domestic ancestry in association with the distribution and history of this lineage. Subcluster **Ie** haplotypes are commonly distributed in other domestic herds within both NAD, KR and Kola Peninsula (Kvie, Heggenes, Anderson, et al., 2016; Kvie, Heggenes, & Røed, 2016; Røed et al., 2008). The near absence of this lineage among modern wild reindeer, as detected here for the large Taĭmyr population, has been also reported for the Belyĭ Island population located directly north of the I͡Amal Peninsula, and also for the Novai͡a Zemli͡a population located north and west of the I͡ANAD (Kvie, Heggenes, Anderson, et al., 2016). The fact that this maternal lineage is not found in contemporary northern wild reindeer populations may suggest that the contemporary Nenets domestic breed has roots toward the south or southwest.

While the diverse CR cluster **I** is suggested to represent a Euro‐Beringian lineage evolved from a Pleistocene population in northern Eurasia (Flagstad & Røed, 2003; Yannic et al., 2014), the evolutionary history of its subclusters is more uncertain. The relatively shallow divergence of **Ie** from other cluster **I** subclusters implies a more recent divergence from the general Euro‐Beringia lineage as also reported for estimates of time since most recent common ancestor for the different cluster **I** subclusters (Kvie, Heggenes, & Røed, 2016). As outlined above, one hypothesis is that small numbers of an exotic animal could have been early imported, were selectively bred beside other habituated local reindeer, and with the help of mutations amplified through an exploding population, their signatures came to dominate the population of domestic reindeer we know today. It is also possible that a strong geophysical event, possibly associated to the Holocene warm period (9,000–5,000 BP, Kaufman et al., 2004), may have isolated this population away from the general Euro‐Beringia lineage, followed by a sudden expansion of the population due to more favorable climate, could explain the history of this lineage. Possible refugees could have been in the Ural Mountains (Salonen et al., 2011) or the Sai͡an Mountains of Southern Siberia (Laufer, 1917; Prokov'ev, 1940).

Although our results may support a Southern Siberian ancestry to the Nenets reindeer breed, which might be just one of several areas from which reindeer herding and domesticated reindeer spread northwards, there is a need for more data before being able to specify the origin point for the non‐native ancestry of this breed. Besides, the use of other genetic markers may imply a more nuanced domestication history. The predominate use of male reindeer for traveling long distances, and for controlled breeding, may have been part of Northern pastoral traditions for hundreds if not thousands of years and evidence of the intermixing of male lines is not necessarily reflected in the maternal genome.

## CONFLICT OF INTEREST

We declare no conflicts of interest.

## AUTHOR CONTRIBUTIONS

**Knut Håkon Røed:** Conceptualization (lead); data curation (lead); formal analysis (lead); funding acquisition (lead); investigation (lead); methodology (lead); project administration (lead); resources (supporting); software (supporting); supervision (supporting); writing‐original draft (lead); writing – review and editing (lead). **Kjersti Kvie:** Data curation (supporting); formal analysis (supporting); resources (supporting); writing‐original draft (supporting); writing – review and editing (supporting). **Robert J Losey:** Resources (supporting); writing‐original draft (supporting); writing – review and editing (supporting). **Pavel A Kositsev:** Resources (equal); validation (supporting); writing‐original draft (supporting); writing – review and editing (supporting). **Anne K Hufthammer:** Data curation (supporting); resources (supporting); validation (supporting); writing‐original draft (supporting); writing – review and editing (supporting). **Mark J Dwyer:** Resources (supporting); writing‐original draft (supporting). **Vasiliy Goncharov:** Resources (supporting); visualization (supporting); writing‐original draft (supporting). **Konstantin B Klokov:** Resources (supporting); writing‐original draft (supporting). **Dimitry V Arzyutov:** Resources (supporting); Writing‐Original Draft (Supporting); Writing‐Review & Editing (supporting). **Andrei Plekhanov:** Resources (supporting); writing‐original draft (supporting). **David George Anderson:** Funding acquisition (supporting); project administration (supporting); resources (supporting); writing‐original draft (supporting); writing – review and editing (equal).

## Supporting information

Appendix S1

## Data Availability

Data for this study will be available in the Sequence Database at the National Centre for Biotechnical Information (NCBI; GenBank ID: MT146049–MT146444).
