# Supplementary material for: Temporal and structural genetic variation in reindeer (Rangifer tarandus) associated with the pastoral transition in Northwestern Siberia
Source: Ecol Evol. 2020 Apr 28;10(17):9060–72. doi: 10.1002/ece3.6314 (PMC7487228; doi:10.1002/ece3.6314)
Supplement: Supplementary file 1 — Appendix S1 [file ECE3-10-9060-s001.docx]

**Appendix 1.**

**The archaeological sites and bone assemble chronology**.

The archaeological samples were from bone assemblages at twelve excavated sites divided into following fifteen sample codes:

Sample code 1, Podcherem (63.52 N, 57.57 E, also called Bol’shai͡a Derevi͡annai͡a), is a rock shelter on the southern shore of Podcherem River, a tributary to the Pechora River in south-eastern part of the Komi Republic. The site is located on the western foothills of the Northern Ural Mountains where the bedrock consists of limestone. The excavated sediment contained bones of a typical late glacial fauna characteristics for the region (Kosintsev 2007; Kuzmina 1971). Four radiocarbon dates on reindeer bones from the site span from 17,000 –12,430 cal BP (Hufthammer pers. com.). All reindeer bones from this site were given an approximate age of 15,000 cal BP.

Sample codes 2-3, Pymva-Shor-I, is located on the tundra in eastern Nenets Autonomous District (ND) some 140 km west of the town Vorkuta in the Pechora drainage basin (67.10 N, 60.51 E). The site is located on a bedrock ledge about 30 m above the present river, just in front of a steep cliff with a small cave. Based on radiocarbon dating the sediment sequence at the site was subdivided into two stratigraphic units; one of Pleistocene age, sample code 2 (c. 20,000-12,000 cal BP) and one Mid-Holocene age, sample code 3 (c. 6,800-5,000 cal BP) (Hufthammer et al. 2019). The reindeer samples successfully analysed in this study were from both units and have been assigned ages of 13,000 and 5,900 cal BP, respectively (Table A1).

Sample codes 4-8, the sites I͡Anganapė-2, 3, and 4, are a set of caves located within the I͡Anganapė mountain range, Syumkev sub-range of the Shchuch’i͡a river basin, within the Polar Urals (67.42 N, 67.50 E). The caves contain large amounts of bones from many species that appeared to have been accumulated there by predators (Golovachev and Smirnov 2009; Kosint͡sev 2009). The material obtained from I͡Anganapė-2 and 3 were each separated in two based on stratigraphic association and radiocarbon dating (Table A1). The material obtained from the upper and lower layer were given an age of 1,800 and 3,500 cal BP for I͡Anganapė-2 (sample code 5 and 4), and 900 and 2700 cal BP for I͡Anganapė-3 (sample code 7 and 6), although the dating for the upper layer of I͡Anganapė-3 varied a good deal (Table A1). The I͡Anganapė-4 samples (sample code 8) were given an age of 1,800 cal BP due to the dating of two of the three samples analyzed here (Table A1).

Sample code 9-10, the sites Ust’-Poluĭ (sample code 9, 66.30 N, 66.38 E) and Zelenai͡a Gorka (sample code 10, 66.33 N, 66.35 E), are both within the contemporary city of Salekhard on the Ob River. With its many specialized artifacts and the absence of any dwellings, Ust’-Poluĭ is probably a ritual sanctuary dating from the Iron Age rather than a traditional settlement site (Gusev and Fedorova 2012). The harness pieces uncovered at Ust’-Poluĭ are interpreted as being strong signs of the presence of domestic reindeer (Gusev et al. 2016; Moshinskai͡a 1953). An age of 1,700 cal BP for this site is based on three radiocarbon dates obtained on reindeer bones prior to this study (Losey et al. 2018). The Zelenai͡a Gorka site is a human settlement with an economy based on hunting and some fishing (Chernet͡sov 1953). The ceramic patterns found here have been used to build a theory about presence of pastoralism unifying a set of sites in the North (Chernet͡sov 1953). Based on dendrochronology, the settlement has been dated from between 12^th^ and 13^th^ centuries (Shii͡atov et al. 2005), as also supported by our radiocarbon dating (Table A1).

Sample code 11 represents the site I͡Arte-6 (68.54 N, 69.57 E) located in the interior of mid-I͡Amal Peninsula along the I͡Uribeĭ River. This study (Table A1) and previous dendrochronological studies (Shii͡atov and Khantemirov 2000; Nomokonova et al. 2018; Anderson et al. 2019) show that the use of this site was during 11^th^ and 12^th^ centuries. Harness pieces, and a type of unique scraper found here are interpreted as signs of the presence of domestic reindeer (Gusev et al. 2016).

Sample code 12-13 includes the sites I͡Uneta-i͡akha-14 (sample code 12, 70.25 N, 68.38 E) and Tiuteĭ-Sale-1 (sample code 13, 71.25 N, 67.45 E), located close to the western coast of I͡Amal Peninsula, were radiocarbon dated to 700 and 800 cal BP respectively, although the dating for I͡Uneta-i͡akha-14 varied with one sample of more recent origin (Table A1). At the Tiuteĭ-Sale-1 site the only matching set of barbed angular gear interpreted as harness pieces for domestic *Rangifer* have been found (R. Losey, field notes).

Sample code 14, Khėkhė-i͡akha-1 (72.53 N, 71.37 E), is located at the far northern coast of I͡Amal Peninsula and is interpreted as the remains of a habitation site, with remains of a dwelling and tools carved out of reindeer antler, dating to the medieval (Plekhanov, field notes). Our dating gives an age of 700 cal BP for Khėkhė-i͡akha-1 (Table A1).

Sample code 15, Khali͡ato-1 (69.01 N, 69.55 E) is located at a lake in the interior of I͡Amal Peninsula near to the mouth of the I͡Uribeĭ River. The ceramic pattern at this site are associated with the 6^th^ and 7^th^ centuries but the form of the graves is associated with the 17^th^ and 18^th^ centuries (Kardash and Sokolkov 2016). Our radiocarbon date confirmed the age of 300 years cal BP (Table A1). Ritual sculptures and a grave found on this site is interpreted as a sign of a ritual site with presence of domestic reindeer (Kardash and Sokolkov 2016).

Table A1. ^14^C and calibrated radiocarbon dates on reindeer samples from excavation sites in I͡Amal-Nenets Autonomous District in Northwestern Siberia.

| Sample ID | Site | Area | Site  code | Lab  code | ^14^C age  BP | Calibrated  BP | Median  probability  BP |
| --- | --- | --- | --- | --- | --- | --- | --- |
| KR-Y-T3053 | I͡Anganapė-2 (UL) | Polar Ural | 5 | Ua-52343 | 1821 ± 30 | 1811-1817 | 1762 |
| KR-Y-T3054 | I͡Anganapė-2 (UL) | Polar Ural | 5 | Ua-52344 | 1894 ± 30 | 1880-1818 | 1844 |
| KR-Y-T3065 | I͡Anganapė-2 (UL) | Polar Ural | 5 | Ua-52345 | 1826 ± 30 | 1812-1725 | 1766 |
| KR-Y-T3066 | I͡Anganapė-2 (UL) | Polar Ural | 5 | Ua-52346 | 1810 ± 29 | 1810-1709 | 1750 |
| KR-Y-T3080 | I͡Anganapė-2 (LL) | Polar Ural | 4 | Ua-52347 | 3183 ± 32 | 3456-3380 | 3409 |
| KR-Y-T3083 | I͡Anganapė-2 (LL) | Polar Ural | 4 | Ua-52348 | 3362 ± 31 | 3638-3572 | 3607 |
| KR-Y-T3088 | I͡Anganapė-3 (UL) | Polar Ural | 7 | Ua-52349 | 1837 ± 32 | 1817-1733 | 1774 |
| KR-Y-T3089 | I͡Anganapė-3 (UL) | Polar Ural | 7 | Ua-52350 | 876 ± 31 | 892-735 | 783 |
| KR-Y-T3095 | I͡Anganapė-3 (UL) | Polar Ural | 7 | Ua-52351 | 142 ± 29 | 272-10 | 145 |
| KR-Y-T3099 | I͡Anganapė-3 (UL) | Polar Ural | 7 | Ua-52352 | 870 ± 30 | 891-732 | 775 |
| KR-Y-T3108 | I͡Anganapė-3 (LL) | Polar Ural | 6 | Ua-52353 | 3636 ± 34 | 4058-3894 | 3894 |
| KR-Y-T3109 | I͡Anganapė-3 (LL) | Polar Ural | 6 | Ua-52354 | 999 ± 30 | 957-834 | 924 |
| KR-Y-T3120 | I͡Anganapė-3 (LL) | Polar Ural | 6 | Ua-52355 | 3120 ± 34 | 3383-3259 | 3341 |
| KR-Y-T3128 | Zelenai͡a Gorka | Salekhard | 10 | Ua-52253 | 739 ± 30 | 690-667 | 681 |
| KR-Y-T3134 | Zelenai͡a Gorka | Salekhard | 10 | Ua-52254 | 642 ± 30 | 659-562 | 599 |
| KR-Y-T3188 | Tiuteĭ-Sale-1 | I͡Amal Peninsula | 13 | Ua-52255 | 618 ± 29 | 651-580 | 601 |
| KR-Y-T3190 | Tiuteĭ-Sale-1 | I͡Amal Peninsula | 13 | Ua-52256 | 618 ± 29 | 651-557 | 601 |
| KR-Y-T3205 | Tiuteĭ-Sale-1 | I͡Amal Peninsula | 13 | Ua-52257 | 798 ± 30 | 729-689 | 713 |
| KR-Y-T3206 | Tiuteĭ-Sale-1 | I͡Amal Peninsula | 13 | Ua-52258 | 1258 ± 30 | 1261-1179 | 1217 |
| KR-Y-T3208 | Khėkhė-i͡akha-1 | I͡Amal Peninsula | 14 | Ua-52259 | 708 ± 31 | 681-656 | 668 |
| KR-Y-T3213 | Khėkhė-i͡akha-1 | I͡Amal Peninsula | 14 | Ua-52260 | 764 ± 30 | 722-673 | 693 |
| KR-Y-T3233 | I͡Anganapė-4 | Polar Ural | 8 | Ua-52261 | 1567 ± 33 | 1521-1412 | 1466 |
| KR-Y-T3236 | I͡Anganapė-4 | Polar Ural | 8 | Ua-52262 | 2095 ± 33 | 2117-2008 | 2068 |
| KR-Y-T3251 | I͡Uneta-i͡akha-14 | Yamal Peninsula | 12 | Ua-52263 | 110 ± 30 | 257-13 | 115 |
| KR-Y-T3254 | I͡Uneta-i͡akha-14 | Yamal Peninsula | 12 | Ua-52264 | 1304 ± 40 | 1285-1185 | 1243 |
| DGA-0/19.01 | I͡Arte-6 | Yamal Peninsula | 11 | Ua-53123 | 981 ± 26 | 932-804 | 901 |
| DGA-0/19.02 | I͡Arte-6 | Yamal Peninsula | 11 | Ua-53124 | 974 ± 27 | 930-803 | 864 |
| DGA-0/19.03 | I͡Arte-6 | Yamal Peninsula | 11 | Ua-53125 | 989 ± 26 | 935-804 | 916 |
| DGA-H/21.01 | I͡Arte-6 | Yamal Peninsula | 11 | Ua-53126 | 996 ± 27 | 954-834 | 923 |
| DGA-H/21.02 | I͡Arte-6 | Yamal Peninsula | 11 | Ua-53127 | 967 ± 26 | 927-802 | 858 |
| DGA-H/21.03 | I͡Arte-6 | Yamal Peninsula | 11 | Ua-53128 | 1001 ± 26 | 955-915 | 927 |
| DGA-H/22.01 | I͡Arte-6 | Yamal Peninsula | 11 | Ua-53129 | 949 ± 26 | 920-825 | 853 |
| DGA-H/2.02 | I͡Arte-6 | Yamal Peninsula | 11 | Ua-53130 | 987 ± 26 | 934-804 | 913 |
| DGA-H/22.03 | I͡Arte-6 | Yamal Peninsula | 11 | Ua-53131 | 985 ± 27 | 934-804 | 909 |
| DGA-M/21.01 | I͡Arte-6 | Yamal Peninsula | 11 | Ua-53132 | 976 ± 26 | 930-803 | 867 |
| DGA-M/21.02 | I͡Arte-6 | Yamal Peninsula | 11 | Ua-53133 | 935 ± 26 | 910-798 | 853 |
| DGA-M/21.03 | I͡Arte-6 | Yamal Peninsula | 11 | Ua-53134 | 982 ± 26 | 932-804 | 904 |
| N632/5 | Khali͡ato-1 | Yamal Peninsula | 15 | Ua-52978 | 257 ± 26 | 311-156 | 301 |
| N632/20 | Khali͡ato-1 | Yamal Peninsula | 15 | Ua-52979 | 243 ± 26 | 305-156 | 291 |

Table A2. Summary of the mtDNA CR-region amplified from archaeological reindeer with subsequent cloning in plasmid vector. The sequences obtained from the clones are compared with the consensus sequence (*cs*) obtained fom the same samples by two independent PCR amplifications.

|  |  | |  | | | | |  | Substitution pattern observed among the misincorporations | | | | | | | |
| --- | --- | --- | --- | --- | --- | --- | --- | --- | --- | --- | --- | --- | --- | --- | --- | --- |
| Sample name | Sample code | Amplicon versus *cs* | | # clones sequenced | # clones different | # clones identical *cs* | # clones identi-cal and ≠ *cs* | C-T | | T-C | G-A | A-G | A-C | G-T | T-G | T-A |
| 1A3064 | 5 | Identical | | 8 | 5 | 4 |  | 4 | |  | 4 |  |  |  | 1 |  |
| 1A3071 | 5 | Identical | | 12 | 8 | 3 | 2 | 7 | |  | 11 | 1 |  |  |  | 1 |
| 6A3192 | 13 | Identical | | 9 | 5 | 5 |  |  | |  |  |  |  |  |  |  |
| 6A3202 | 13 | Identical | | 12 | 10 | 3 |  | 20 | |  | 7 |  |  |  |  |  |
| 7A3209 | 14 | Identical | | 11 | 3 | 8 | 2 | 1 | |  | 1 |  |  |  |  |  |
| 8A3219 | 15 | Identical | | 7 | 4 | 3 | 2 | 2 | |  | 1 |  |  |  |  |  |
| 8A3217 | 15 | Identical | | 10 | 6 | 4 | 2 | 6 | |  | 2 |  |  |  |  |  |
| 8A3218 | 15 | Identical | | 10 | 5 | 6 |  | 3 | |  | 2 |  |  |  |  |  |
| AA3233 | 8 | Identical | | 10 | 6 | 5 |  |  | | 1 | 5 |  |  |  |  |  |
| AA3236 | 8 | Identical | | 11 | 5 | 7 |  | 4 | |  | 2 |  |  |  |  |  |
| AA3237 | 8 | Identical | | 9 | 6 | 4 |  | 5 | |  |  |  | 1 | 1 |  |  |
| 9A3247 | 12 | Identical | | 11 | 5 | 6 | 2 | 7 | | 2 | 2 | 3 |  |  |  |  |
| 1A3072 | 5 | Identical | | 10 | 4 | 5 | 2 | 2 | |  | 2 |  |  |  |  |  |
| 3A3095 | 7 | Identical | | 9 | 3 | 7 |  | 3 | | 1 | 1 |  |  |  |  |  |
| 5A3179 | 11 | Identical | | 9 | 6 | 2 | 2 | 8 | | 1 | 8 | 2 |  |  |  |  |
| 5A3178 | 11 | Identical | | 10 | 6 | 5 |  | 8 | |  | 3 | 1 |  |  |  |  |
| 9A3246 | 12 | Identical | | 6 | 5 | 2 |  | 3 | | 1 | 2 |  |  |  |  | 1 |
| 3A3096 | 7 | Identical | | 10 | 9 | 2 |  | 17 | | 1 | 7 |  |  |  |  |  |
| 3A3112 | 6 | Identical | | 12 | 12 | 0 |  | 29 | | 1 | 4 |  |  |  |  |  |
| 9A3245 | 12 | Identical | | 13 | 12 | 1 | 2 | 20 | | 7 | 3 |  |  |  |  |  |

Tables A3. Sampling locations, sequence names and GenBank accession numbers for the control region (CR) in archaic reindeer (sample codes 1 – 15), contemporary domestic (sample codes 16 - 19) and wild reindeer (sample code 20) in Northwestern Siberia.

| Sample  location | Sample code | Sequence name | Accession number |
| --- | --- | --- | --- |
| Podcherem | 1 | AncRt2347 | MT146056 |
| Podcherem | 1 | AncRt2348 | MT146057 |
| Podcherem | 1 | AncRt2349 | MT146058 |
| Podcherem | 1 | AncRt2350 | MT146059 |
| Podcherem | 1 | AncRt2354 | MT146060 |
| Podcherem | 1 | AncRt2355 | MT146061 |
| Podcherem | 1 | AncRt2356 | MT146062 |
| Podcherem | 1 | AncRt2357 | MT146063 |
| Podcherem | 1 | AncRt2360 | MT146064 |
| Podcherem | 1 | AncRt2361 | MT146065 |
| Podcherem | 1 | AncRt2362 | MT146066 |
| Podcherem | 1 | AncRt2363 | MT146067 |
| Podcherem | 1 | AncRt2364 | MT146068 |
| Podcherem | 1 | AncRt2365 | MT146069 |
| Podcherem | 1 | AncRt2367 | MT146070 |
| Podcherem | 1 | AncRt2369 | MT146071 |
| Podcherem | 1 | AncRt2370 | MT146072 |
| Podcherem | 1 | AncRt2371 | MT146073 |
| Podcherem | 1 | AncRt2374 | MT146074 |
| Pymva-Shor (LL) | 2 | AncRt1837 | MT146075 |
| Pymva-Shor (LL) | 2 | AncRt1848 | MT146078 |
| Pymva-Shor (UL) | 3 | AncRt1842 | MT146076 |
| Pymva-Shor (UL) | 3 | AncRt1845 | MT146077 |
| Pymva-Shor (UL) | 3 | AncRt1850 | MT146079 |
| Pymva-Shor (UL) | 3 | AncRt1851 | MT146080 |
| Pymva-Shor (UL) | 3 | AncRt1852 | MT146081 |
| Pymva-Shor (UL) | 3 | AncRt1853 | MT146082 |
| Pymva-Shor (UL) | 3 | AncRt1854 | MT146083 |
| Pymva-Shor (UL) | 3 | AncRt1861 | MT146084 |
| Pymva-Shor (UL) | 3 | AncRt1862 | MT146085 |
| Pymva-Shor (UL) | 3 | AncRt1865 | MT146086 |
| Pymva-Shor (UL) | 3 | AncRt219 | MT146087 |
| Pymva-Shor (UL) | 3 | AncRt221 | MT146088 |
| Pymva-Shor (UL) | 3 | AncRt224 | MT146089 |
| Pymva-Shor (UL) | 3 | AncRt226 | MT146090 |
| I͡Anganapė-2 (LL) | 4 | AncRt3076 | MT146114 |
| I͡Anganapė-2 (LL) | 4 | AncRt3078 | MT146115 |
| I͡Anganapė-2 (LL) | 4 | AncRt3080 | MT146116 |
| I͡Anganapė-2 (LL) | 4 | AncRt3082 | MT146117 |
| I͡Anganapė-2 (LL) | 4 | AncRt3083 | MT146118 |
| I͡Anganapė-2 (LL) | 4 | AncRt3084 | MT146119 |
| I͡Anganapė-2 (UL) | 5 | AncRt3047 | MT146091 |
| I͡Anganapė-2 (UL) | 5 | AncRt3050 | MT146092 |
| I͡Anganapė-2 (UL) | 5 | AncRt3051 | MT146093 |
| I͡Anganapė-2 (UL) | 5 | AncRt3052 | MT146094 |
| I͡Anganapė-2 (UL) | 5 | AncRt3053 | MT146095 |
| I͡Anganapė-2 (UL) | 5 | AncRt3054 | MT146096 |
| I͡Anganapė-2 (UL) | 5 | AncRt3055 | MT146097 |
| I͡Anganapė-2 (UL) | 5 | AncRt3056 | MT146098 |
| I͡Anganapė-2 (UL) | 5 | AncRt3057 | MT146099 |
| I͡Anganapė-2 (UL) | 5 | AncRt3059 | MT146100 |
| I͡Anganapė-2 (UL) | 5 | AncRt3060 | MT146101 |
| I͡Anganapė-2 (UL) | 5 | AncRt3062 | MT146102 |
| I͡Anganapė-2 (UL) | 5 | AncRt3063 | MT146103 |
| I͡Anganapė-2 (UL) | 5 | AncRt3064 | MT146104 |
| I͡Anganapė-2 (UL) | 5 | AncRt3065 | MT146105 |
| I͡Anganapė-2 (UL) | 5 | AncRt3066 | MT146106 |
| I͡Anganapė-2 (UL) | 5 | AncRt3067 | MT146107 |
| I͡Anganapė-2 (UL) | 5 | AncRt3068 | MT146108 |
| I͡Anganapė-2 (UL) | 5 | AncRt3069 | MT146109 |
| I͡Anganapė-2 (UL) | 5 | AncRt3071 | MT146110 |
| I͡Anganapė-2 (UL) | 5 | AncRt3072 | MT146111 |
| I͡Anganapė-2 (UL) | 5 | AncRt3073 | MT146112 |
| I͡Anganapė-2 (UL) | 5 | AncRt3074 | MT146113 |
| I͡Anganapė-3 (LL) | 6 | AncRt3105 | MT146139 |
| I͡Anganapė-3 (LL) | 6 | AncRt3106 | MT146140 |
| I͡Anganapė-3 (LL) | 6 | AncRt3107 | MT146141 |
| I͡Anganapė-3 (LL) | 6 | AncRt3108 | MT146142 |
| I͡Anganapė-3 (LL) | 6 | AncRt3109 | MT146143 |
| I͡Anganapė-3 (LL) | 6 | AncRt3110 | MT146144 |
| I͡Anganapė-3 (LL) | 6 | AncRt3111 | MT146145 |
| I͡Anganapė-3 (LL) | 6 | AncRt3112 | MT146146 |
| I͡Anganapė-3 (LL) | 6 | AncRt3113 | MT146147 |
| I͡Anganapė-3 (LL) | 6 | AncRt3115 | MT146148 |
| I͡Anganapė-3 (LL) | 6 | AncRt3116 | MT146149 |
| I͡Anganapė-3 (LL) | 6 | AncRt3117 | MT146150 |
| I͡Anganapė-3 (LL) | 6 | AncRt3118 | MT146151 |
| I͡Anganapė-3 (LL) | 6 | AncRt3119 | MT146152 |
| I͡Anganapė-3 (LL) | 6 | AncRt3120 | MT146153 |
| I͡Anganapė-3 (LL) | 6 | AncRt3121 | MT146154 |
| I͡Anganapė-3 (LL) | 6 | AncRt3122 | MT146155 |
| I͡Anganapė-3 (LL) | 6 | AncRt3123 | MT146156 |
| I͡Anganapė-3 (LL) | 6 | AncRt3124 | MT146157 |
| I͡Anganapė-3 (LL) | 6 | AncRt3126 | MT146158 |
| I͡Anganapė-3 (LL) | 6 | AncRt3127 | MT146159 |
| I͡Anganapė-3 (UL) | 7 | AncRt3085 | MT146120 |
| I͡Anganapė-3 (UL) | 7 | AncRt3087 | MT146121 |
| I͡Anganapė-3 (UL) | 7 | AncRt3088 | MT146122 |
| I͡Anganapė-3 (UL) | 7 | AncRt3089 | MT146123 |
| I͡Anganapė-3 (UL) | 7 | AncRt3090 | MT146124 |
| I͡Anganapė-3 (UL) | 7 | AncRt3091 | MT146125 |
| I͡Anganapė-3 (UL) | 7 | AncRt3092 | MT146126 |
| I͡Anganapė-3 (UL) | 7 | AncRt3093 | MT146127 |
| I͡Anganapė-3 (UL) | 7 | AncRt3094 | MT146128 |
| I͡Anganapė-3 (UL) | 7 | AncRt3095 | MT146129 |
| I͡Anganapė-3 (UL) | 7 | AncRt3096 | MT146130 |
| I͡Anganapė-3 (UL) | 7 | AncRt3097 | MT146131 |
| I͡Anganapė-3 (UL) | 7 | AncRt3098 | MT146132 |
| I͡Anganapė-3 (UL) | 7 | AncRt3099 | MT146133 |
| I͡Anganapė-3 (UL) | 7 | AncRt3100 | MT146134 |
| I͡Anganapė-3 (UL) | 7 | AncRt3101 | MT146135 |
| I͡Anganapė-3 (UL) | 7 | AncRt3102 | MT146136 |
| I͡Anganapė-3 (UL) | 7 | AncRt3103 | MT146137 |
| I͡Anganapė-3 (UL) | 7 | AncRt3104 | MT146138 |
| I͡Anganapė-4 | 8 | AncRt3233 | MT146260 |
| I͡Anganapė-4 | 8 | AncRt3236 | MT146261 |
| I͡Anganapė-4 | 8 | AncRt3237 | MT146262 |
| Ust’-Poluĭ | 9 | AncRt3238 | MT146263 |
| Zelenai͡a Gorka | 10 | AncRt3128 | MT146160 |
| Zelenai͡a Gorka | 10 | AncRt3129 | MT146161 |
| Zelenai͡a Gorka | 10 | AncRt3130 | MT146162 |
| Zelenai͡a Gorka | 10 | AncRt3131 | MT146163 |
| Zelenai͡a Gorka | 10 | AncRt3132 | MT146164 |
| Zelenai͡a Gorka | 10 | AncRt3133 | MT146165 |
| Zelenai͡a Gorka | 10 | AncRt3134 | MT146166 |
| Zelenai͡a Gorka | 10 | AncRt3135 | MT146167 |
| Zelenai͡a Gorka | 10 | AncRt3136 | MT146168 |
| Zelenai͡a Gorka | 10 | AncRt3137 | MT146169 |
| Zelenai͡a Gorka | 10 | AncRt3138 | MT146170 |
| Zelenai͡a Gorka | 10 | AncRt3139 | MT146171 |
| Zelenai͡a Gorka | 10 | AncRt3140 | MT146172 |
| Zelenai͡a Gorka | 10 | AncRt3142 | MT146173 |
| Zelenai͡a Gorka | 10 | AncRt3143 | MT146174 |
| Zelenai͡a Gorka | 10 | AncRt3145 | MT146175 |
| Zelenai͡a Gorka | 10 | AncRt3147 | MT146176 |
| Zelenai͡a Gorka | 10 | AncRt3148 | MT146177 |
| Zelenai͡a Gorka | 10 | AncRt3150 | MT146178 |
| Zelenai͡a Gorka | 10 | AncRt3151 | MT146179 |
| Zelenai͡a Gorka | 10 | AncRt3152 | MT146180 |
| Zelenai͡a Gorka | 10 | AncRt3153 | MT146181 |
| Zelenai͡a Gorka | 10 | AncRt3154 | MT146182 |
| I͡Arte-6 | 11 | AncRt3155 | MT146183 |
| I͡Arte-6 | 11 | AncRt3156 | MT146184 |
| I͡Arte-6 | 11 | AncRt3157 | MT146185 |
| I͡Arte-6 | 11 | AncRt3158 | MT146186 |
| I͡Arte-6 | 11 | AncRt3159 | MT146187 |
| I͡Arte-6 | 11 | AncRt3160 | MT146188 |
| I͡Arte-6 | 11 | AncRt3161 | MT146189 |
| I͡Arte-6 | 11 | AncRt3162 | MT146190 |
| I͡Arte-6 | 11 | AncRt3163 | MT146191 |
| I͡Arte-6 | 11 | AncRt3164 | MT146192 |
| I͡Arte-6 | 11 | AncRt3165 | MT146193 |
| I͡Arte-6 | 11 | AncRt3166 | MT146194 |
| I͡Arte-6 | 11 | AncRt3167 | MT146195 |
| I͡Arte-6 | 11 | AncRt3168 | MT146196 |
| I͡Arte-6 | 11 | AncRt3169 | MT146197 |
| I͡Arte-6 | 11 | AncRt3171 | MT146198 |
| I͡Arte-6 | 11 | AncRt3172 | MT146199 |
| I͡Arte-6 | 11 | AncRt3173 | MT146200 |
| I͡Arte-6 | 11 | AncRt3174 | MT146201 |
| I͡Arte-6 | 11 | AncRt3175 | MT146202 |
| I͡Arte-6 | 11 | AncRt3176 | MT146203 |
| I͡Arte-6 | 11 | AncRt3177 | MT146204 |
| I͡Arte-6 | 11 | AncRt3178 | MT146205 |
| I͡Arte-6 | 11 | AncRt3179 | MT146206 |
| I͡Arte-6 | 11 | AncRt3180 | MT146207 |
| I͡Arte-6 | 11 | AncRt3181 | MT146208 |
| I͡Arte-6 | 11 | AncRt3182 | MT146209 |
| I͡Arte-6 | 11 | AncRt3183 | MT146210 |
| I͡Arte-6 | 11 | AncRt3184 | MT146211 |
| I͡Arte-6 | 11 | AncRt3277 | MT146212 |
| I͡Arte-6 | 11 | AncRt3278 | MT146213 |
| I͡Arte-6 | 11 | AncRt3280 | MT146214 |
| I͡Arte-6 | 11 | AncRt3281 | MT146215 |
| I͡Arte-6 | 11 | AncRt3282 | MT146216 |
| I͡Arte-6 | 11 | AncRt3283 | MT146217 |
| I͡Arte-6 | 11 | AncRt3284 | MT146218 |
| I͡Arte-6 | 11 | AncRt3585 | MT146219 |
| I͡Arte-6 | 11 | AncRt3286 | MT146220 |
| I͡Arte-6 | 11 | AncRt3287 | MT146221 |
| I͡Arte-6 | 11 | AncRt3288 | MT146222 |
| I͡Arte-6 | 11 | AncRt3289 | MT146223 |
| I͡Uneta-i͡akha-14 | 12 | AncRt3241 | MT146264 |
| I͡Uneta-i͡akha-14 | 12 | AncRt3242 | MT146265 |
| I͡Uneta-i͡akha-14 | 12 | AncRt3243 | MT146266 |
| I͡Uneta-i͡akha-14 | 12 | AncRt3245 | MT146267 |
| I͡Uneta-i͡akha-14 | 12 | AncRt3246 | MT146268 |
| I͡Uneta-i͡akha-14 | 12 | AncRt3247 | MT146269 |
| I͡Uneta-i͡akha-14 | 12 | AncRt3248 | MT146270 |
| I͡Uneta-i͡akha-14 | 12 | AncRt3249 | MT146271 |
| I͡Uneta-i͡akha-14 | 12 | AncRt3250 | MT146272 |
| I͡Uneta-i͡akha-14 | 12 | AncRt3251 | MT146273 |
| I͡Uneta-i͡akha-14 | 12 | AncRt3252 | MT146274 |
| I͡Uneta-i͡akha-14 | 12 | AncRt3253 | MT146275 |
| I͡Uneta-i͡akha-14 | 12 | AncRt3254 | MT146276 |
| I͡Uneta-i͡akha-14 | 12 | AncRt3255 | MT146277 |
| I͡Uneta-i͡akha-14 | 12 | AncRt3256 | MT146278 |
| Tiuteĭ -Sale-1 | 13 | AncRt3187 | MT146224 |
| Tiuteĭ -Sale-1 | 13 | AncRt3190 | MT146225 |
| Tiuteĭ -Sale-1 | 13 | AncRt3192 | MT146226 |
| Tiuteĭ -Sale-1 | 13 | AncRt3193 | MT146227 |
| Tiuteĭ -Sale-1 | 13 | AncRt3195 | MT146228 |
| Tiuteĭ -Sale-1 | 13 | AncRt3196 | MT146229 |
| Tiuteĭ -Sale-1 | 13 | AncRt3197 | MT146230 |
| Tiuteĭ -Sale-1 | 13 | AncRt3199 | MT146231 |
| Tiuteĭ -Sale-1 | 13 | AncRt3200 | MT146232 |
| Tiuteĭ -Sale-1 | 13 | AncRt3202 | MT146233 |
| Tiuteĭ -Sale-1 | 13 | AncRt3203 | MT146234 |
| Tiuteĭ -Sale-1 | 13 | AncRt3204 | MT146235 |
| Tiuteĭ -Sale-1 | 13 | AncRt3205 | MT146236 |
| Tiuteĭ -Sale-1 | 13 | AncRt3206 | MT146237 |
| Tiuteĭ -Sale-1 | 13 | AncRt3207 | MT146238 |
| Khėkhė-i͡akha-1 | 14 | AncRt3209 | MT146239 |
| Khėkhė-i͡akha-1 | 14 | AncRt3210 | MT146240 |
| Khėkhė-i͡akha-1 | 14 | AncRt3211 | MT146241 |
| Khėkhė-i͡akha-1 | 14 | AncRt3212 | MT146242 |
| Khėkhė-i͡akha-1 | 14 | AncRt3213 | MT146243 |
| Khėkhė-i͡akha-1 | 14 | AncRt3214 | MT146244 |
| Khėkhė-i͡akha-1 | 14 | AncRt3215 | MT146245 |
| Khėkhė-i͡akha-1 | 14 | AncRt3216 | MT146246 |
| Khali͡ato-1 | 15 | AncRt3217 | MT146247 |
| Khali͡ato-1 | 15 | AncRt3218 | MT146248 |
| Khali͡ato-1 | 15 | AncRt3219 | MT146249 |
| Khali͡ato-1 | 15 | AncRt3220 | MT146250 |
| Khali͡ato-1 | 15 | AncRt3221 | MT146251 |
| Khali͡ato-1 | 15 | AncRt3222 | MT146252 |
| Khali͡ato-1 | 15 | AncRt3223 | MT146253 |
| Khali͡ato-1 | 15 | AncRt3224 | MT146254 |
| Khali͡ato-1 | 15 | AncRt3225 | MT146255 |
| Khali͡ato-1 | 15 | AncRt3226 | MT146256 |
| Khali͡ato-1 | 15 | AncRt3227 | MT146257 |
| Khali͡ato-1 | 15 | AncRt3928 | MT146258 |
| Khali͡ato-1 | 15 | AncRt3229 | MT146259 |
| I͡Amal South | 16 | Re4675 | MT146315 |
| I͡Amal South | 16 | Re4676 | MT146316 |
| I͡Amal South | 16 | Re4677 | MT146317 |
| I͡Amal South | 16 | Re4678 | MT146318 |
| I͡Amal South | 16 | Re4679 | MT146319 |
| I͡Amal South | 16 | Re4680 | MT146320 |
| I͡Amal South | 16 | Re4681 | MT146321 |
| I͡Amal South | 16 | Re4682 | MT146322 |
| I͡Amal South | 16 | Re4683 | MT146323 |
| I͡Amal South | 16 | Re4684 | MT146324 |
| I͡Amal South | 16 | Re4685 | MT146325 |
| I͡Amal South | 16 | Re4686 | MT146326 |
| I͡Amal South | 16 | Re4687 | MT146327 |
| I͡Amal South | 16 | Re4688 | MT146328 |
| I͡Amal South | 16 | Re4689 | MT146329 |
| I͡Amal South | 16 | Re4690 | MT146330 |
| I͡Amal South | 16 | Re4691 | MT146331 |
| I͡Amal South | 16 | Re4692 | MT146332 |
| I͡Amal South | 16 | Re4693 | MT146333 |
| I͡Amal South | 16 | Re4694 | MT146334 |
| I͡Amal South | 16 | Re4695 | MT146335 |
| I͡Amal South | 16 | Re4696 | MT146336 |
| I͡Amal South | 16 | Re4697 | MT146337 |
| I͡Amal South | 16 | Re4698 | MT146338 |
| I͡Amal South | 16 | Re4699 | MT146339 |
| I͡Amal South | 16 | Re4700 | MT146340 |
| I͡Amal South | 16 | Re4701 | MT146341 |
| I͡Amal South | 16 | Re5023 | MT146342 |
| I͡Amal South | 16 | Re5024 | MT146343 |
| I͡Amal South | 16 | Re5025 | MT146344 |
| I͡Amal South | 16 | Re5026 | MT146345 |
| I͡Amal South | 16 | Re5027 | MT146346 |
| I͡Amal South | 16 | Re5028 | MT146347 |
| I͡Amal South | 16 | Re5029 | MT146348 |
| I͡Amal South | 16 | Re5762 | MT146349 |
| I͡Amal South | 16 | Re5763 | MT146350 |
| I͡Amal South | 16 | Re5766 | MT146351 |
| I͡Amal South | 16 | Re5768 | MT146352 |
| I͡Amal South | 16 | Re5769 | MT146353 |
| I͡Amal South | 16 | Re5770 | MT146354 |
| I͡Amal South | 16 | Re5771 | MT146355 |
| I͡Amal South | 16 | Re5773 | MT146356 |
| I͡Amal South | 16 | Re5774 | MT146357 |
| I͡Amal South | 16 | Re5775 | MT146358 |
| I͡Amal South | 16 | Re5776 | MT146359 |
| I͡Amal South | 16 | Re5777 | MT146360 |
| I͡Amal South | 16 | Re5778 | MT146361 |
| I͡Amal South | 16 | Re5781 | MT146362 |
| I͡Amal South | 16 | Re5782 | MT146363 |
| I͡Amal South | 16 | Re5784 | MT146364 |
| I͡Amal South | 16 | Re5785 | MT146365 |
| I͡Amal South | 16 | Re5447 | MT146366 |
| I͡Amal South | 16 | Re5448 | MT146367 |
| I͡Amal South | 16 | Re5449 | MT146368 |
| I͡Amal South | 16 | Re5450 | MT146369 |
| I͡Amal South | 16 | Re5451 | MT146370 |
| I͡Amal North | 17 | Re5013 | MT146279 |
| I͡Amal North | 17 | Re5014 | MT146280 |
| I͡Amal North | 17 | Re5015 | MT146281 |
| I͡Amal North | 17 | Re5016 | MT146282 |
| I͡Amal North | 17 | Re5017 | MT146283 |
| I͡Amal North | 17 | Re5018 | MT146284 |
| I͡Amal North | 17 | Re5019 | MT146285 |
| I͡Amal North | 17 | Re5020 | MT146286 |
| I͡Amal North | 17 | Re5021 | MT146287 |
| I͡Amal North | 17 | Re6261 | MT146288 |
| I͡Amal North | 17 | Re6263 | MT146289 |
| I͡Amal North | 17 | Re6265 | MT146290 |
| I͡Amal North | 17 | Re6266 | MT146291 |
| I͡Amal North | 17 | Re6267 | MT146292 |
| I͡Amal North | 17 | Re6268 | MT146293 |
| I͡Amal North | 17 | Re6269 | MT146294 |
| I͡Amal North | 17 | Re6270 | MT146295 |
| I͡Amal North | 17 | Re6272 | MT146296 |
| I͡Amal North | 17 | Re6275 | MT146297 |
| I͡Amal North | 17 | Re6276 | MT146298 |
| I͡Amal North | 17 | Re6277 | MT146299 |
| I͡Amal North | 17 | Re5445 | MT146300 |
| I͡Amal North | 17 | Re5446 | MT146301 |
| Taz-Nenets | 18 | Re4207 | MT146302 |
| Taz-Nenets | 18 | Re4214 | MT146303 |
| Taz-Nenets | 18 | Re4225 | MT146304 |
| Taz-Nenets | 18 | Re4226 | MT146305 |
| Taz-Nenets | 18 | Re4227 | MT146306 |
| Taz-Nenets | 18 | Re4232 | MT146307 |
| Taz-Nenets | 18 | Re4237 | MT146308 |
| Taz-Nenets | 18 | Re4239 | MT146309 |
| Taz-Nenets | 18 | Re4241 | MT146310 |
| Taz-Nenets | 18 | Re4244 | MT146311 |
| Taz-Nenets | 18 | Re4245 | MT146312 |
| Taz-Nenets | 18 | Re4248 | MT146313 |
| Taz-Nenets | 18 | Re4250 | MT146314 |
| Eniseĭ-Nenets | 19 | Re6539 | MT146371 |
| Eniseĭ-Nenets | 19 | Re6540 | MT146372 |
| Eniseĭ-Nenets | 19 | Re6541 | MT146373 |
| Eniseĭ-Nenets | 19 | Re6542 | MT146374 |
| Eniseĭ-Nenets | 19 | Re6543 | MT146375 |
| Eniseĭ-Nenets | 19 | Re6544 | MT146376 |
| Eniseĭ-Nenets | 19 | Re6545 | MT146377 |
| Eniseĭ-Nenets | 19 | Re6546 | MT146378 |
| Eniseĭ-Nenets | 19 | Re6547 | MT146379 |
| Eniseĭ-Nenets | 19 | Re6548 | MT146380 |
| Eniseĭ-Nenets | 19 | Re6549 | MT146381 |
| Eniseĭ-Nenets | 19 | Re6553 | MT146382 |
| Eniseĭ-Nenets | 19 | Re6554 | MT146383 |
| Eniseĭ-Nenets | 19 | Re6555 | MT146384 |
| Eniseĭ-Nenets | 19 | Re6556 | MT146385 |
| Eniseĭ-Nenets | 19 | Re6557 | MT146386 |
| Eniseĭ-Nenets | 19 | Re6558 | MT146387 |
| Eniseĭ-Nenets | 19 | Re6559 | MT146388 |
| Eniseĭ-Nenets | 19 | Re6560 | MT146389 |
| Eniseĭ-Nenets | 19 | Re6561 | MT146390 |
| Eniseĭ-Nenets | 19 | Re6562 | MT146391 |
| Eniseĭ-Nenets | 19 | Re6563 | MT146392 |
| Eniseĭ-Nenets | 19 | Re6564 | MT146393 |
| Eniseĭ-Nenets | 19 | Re6565 | MT146394 |
| Eniseĭ-Nenets | 19 | Re6566 | MT146395 |
| Eniseĭ-Nenets | 19 | Re6568 | MT146396 |
| Eniseĭ-Nenets | 19 | Re6571 | MT146397 |
| Eniseĭ-Nenets | 19 | Re6572 | MT146398 |
| Eniseĭ-Nenets | 19 | Re6573 | MT146399 |
| Eniseĭ-Nenets | 19 | Re6576 | MT146400 |
| Eniseĭ-Nenets | 19 | Re6577 | MT146401 |
| Eniseĭ-Nenets | 19 | Re6578 | MT146402 |
| Eniseĭ-Nenets | 19 | Re6531 | MT146403 |
| Eniseĭ-Nenets | 19 | Re6532 | MT146404 |
| Eniseĭ-Nenets | 19 | Re6533 | MT146405 |
| Eniseĭ-Nenets | 19 | Re6535 | MT146406 |
| Eniseĭ-Nenets | 19 | Re6536 | MT146407 |
| Eniseĭ-Nenets | 19 | Re6537 | MT146408 |
| Eniseĭ-Nenets | 19 | Re6824 | MT146409 |
| Eniseĭ-Nenets | 19 | Re6826 | MT146410 |
| Eniseĭ-Nenets | 19 | Re6827 | MT146411 |
| Eniseĭ-Nenets | 19 | Re6828 | MT146412 |
| Eniseĭ-Nenets | 19 | Re6829 | MT146413 |
| Eniseĭ-Nenets | 19 | Re6830 | MT146414 |
| Eniseĭ-Nenets | 19 | Re6831 | MT146415 |
| Taymyr | 20 | Re5276 | MT146049 |
| Taymyr | 20 | Re5291 | MT146050 |
| Taymyr | 20 | Re5294 | MT146051 |
| Taymyr | 20 | Re5301 | MT146052 |
| Taymyr | 20 | Re5302 | MT146053 |
| Taymyr | 20 | Re5304 | MT146054 |
| Taymyr | 20 | Re5309 | MT146055 |
| Taymyr | 20 | Re6800 | MT146416 |
| Taymyr | 20 | Re6802 | MT146417 |
| Taymyr | 20 | Re6807 | MT146418 |
| Taymyr | 20 | Re6808 | MT146419 |
| Taymyr | 20 | Re6822 | MT146420 |
| Taymyr | 20 | Re6845 | MT146421 |
| Taymyr | 20 | Re6846 | MT146422 |
| Taymyr | 20 | Re6847 | MT146423 |
| Taymyr | 20 | Re6850 | MT146424 |
| Taymyr | 20 | Re6851 | MT146425 |
| Taymyr | 20 | Re6852 | MT146426 |
| Taymyr | 20 | Re6853 | MT146427 |
| Taymyr | 20 | Re6854 | MT146428 |
| Taymyr | 20 | Re6855 | MT146429 |
| Taymyr | 20 | Re6856 | MT146430 |
| Taymyr | 20 | Re6857 | MT146431 |
| Taymyr | 20 | Re6858 | MT146432 |
| Taymyr | 20 | Re6859 | MT146433 |
| Taymyr | 20 | Re6860 | MT146434 |
| Taymyr | 20 | Re6861 | MT146435 |
| Taymyr | 20 | Re6862 | MT146436 |
| Taymyr | 20 | Re6863 | MT146437 |
| Taymyr | 20 | Re6865 | MT146438 |
| Taymyr | 20 | Re6866 | MT146439 |
| Taymyr | 20 | Re6867 | MT146440 |
| Taymyr | 20 | Re6868 | MT146441 |
| Taymyr | 20 | Re6869 | MT146442 |
| Taymyr | 20 | Re6870 | MT146443 |
| Taymyr | 20 | Re6871 | MT146444 |
| Taymyr | 20 | Re5277 | KX094725 |
| Taymyr | 20 | Re5278 | KX094726 |
| Taymyr | 20 | Re5279 | KX094727 |
| Taymyr | 20 | Re5280 | KX094728 |
| Taymyr | 20 | Re5281 | KX094729 |
| Taymyr | 20 | Re5282 | KX094730 |
| Taymyr | 20 | Re5283 | KX094731 |
| Taymyr | 20 | Re5284 | KX094732 |
| Taymyr | 20 | Re5285 | KX094733 |
| Taymyr | 20 | Re5286 | KX094734 |
| Taymyr | 20 | Re5287 | KX094735 |
| Taymyr | 20 | Re5288 | KX094736 |
| Taymyr | 20 | Re5289 | KX094737 |
| Taymyr | 20 | Re5290 | KX094738 |
| Taymyr | 20 | Re5292 | KX094739 |
| Taymyr | 20 | Re5293 | KX094740 |
| Taymyr | 20 | Re5295 | KX094741 |
| Taymyr | 20 | Re5296 | KX094742 |
| Taymyr | 20 | Re5297 | KX094743 |
| Taymyr | 20 | Re5298 | KX094744 |
| Taymyr | 20 | Re5299 | KX094745 |
| Taymyr | 20 | Re5300 | KX094746 |
| Taymyr | 20 | Re5303 | KX094747 |
| Taymyr | 20 | Re5305 | KX094748 |

Figure A1. Summary of the results of spatial analysis of molecular variance (SAMOVA) using the control region of mtDNA in archaic reindeer (sample code 1 – 15), contemporary domestic (sample code 16 – 19) and wild reindeer (sample code 20) in Northwestern Siberia. The group compositions for the best clustering option of FCT, associated with each predefined value of K (K=2-8) is shown below the plot. Numbers within frames refers to sample codes as given in Table 1 and Fig. 1 in main document.


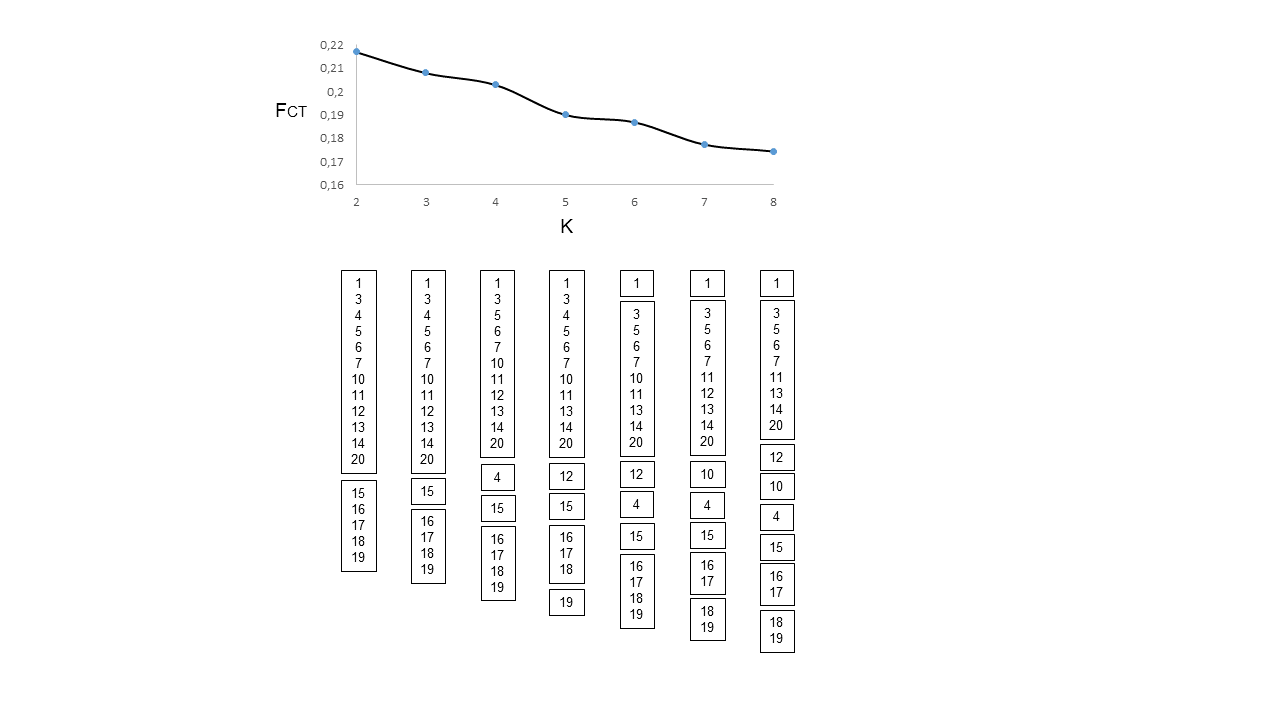


REFERENCES

Anderson, D. G., L. Harrault, K. B. Milek, B. C. Forbes, M. Kuoppamaa, and A. V. Plekhanov. 1919. Animal domestication in the high Arctic: Hunting and holding reindeer on the I͡Amal peninsula, northwest Siberia. *Journal of Anthropological Archaeology* 55, 101079.

Chernet͡sov, V. N. 1953. Nizhnee Priob'e v I tysi͡acheletii nasheĭ ėry. Pages 7-71 *in* V. N. Chernet͡sov, V. I. Moshinskai͡a, and I. A. Talit͡skai͡a, editors, *Drevni͡ai͡a istorii͡a nizhnego Priob'i͡a*. Izd. Akademii nauk, Moscow, Russia.

Golovachev, I. B., and N. G. Smirnov. 2009. The Late Pleistocene and Holocene rodents of the Pre-Urals subarctic. *Quaternary International* 201:37-42.

Gusev, A. V., and N. V. Fedorova. 2012*. Drevnee svi͡atilishche Ust'-Poluĭ: konstrukt͡sii, deĭstvii͡a, artefakty. Itogi issledovaniĭ planigrafii i stratigrafii pami͡atnika: 1935-2012 gg.* GU I͡ANAO "Severnoe izdatel'stvo", Salekhard, Russia.

Gusev, A. V., A. V. Plekhanov, and N. V. Fedorova. 2016. Olenevodstvo na Severe Zapadnoĭ Sibiri: ranniĭ zheleznyĭ vek – Srednevekov'e. Pages 228-239 *in* D. S. Tupakhin, and N. V. Fedorova, editors. *Arkheologii͡a Arktiki*. ROS-DOAFK, Kalingrad, Russia.

Hufthammer, A. K., J. I. Svendsen, and P. Pavlov. 2018. Animals and humans in the European Russian Arctic towards the end of the last Ice Age and during the mid-Holocene time. *Boreas* 48:387-406.

Kardash, O. V., and A. V. Sokolkov. 2016. Ritual'nyĭ kompleks Kholi͡ato-1 na poluostrove I͡Amal. *Arkheologii͡a, ėtnografii͡a i antropologii͡a Evrazii* 43:81-91.

Kosintsev, P. 2007. Late Pleistocene large mammal faunas from the Urals. *Quaternary International* 160:112–120.

Kosint͡sev, P. A. 2009. Pogrebennoe logovo volka na Poli͡arnom Urale. Pages 108-118 *in* N. V. E. Martynovich, editor. *Eniseĭskai͡a provint͡sii͡a. Al'manakh, vypusk*. Krasnoi͡arskiĭ kraevoĭ kraevedcheskiĭ muzeĭ, Krasnoi͡ars, Russia.

Kuzmina, I. E. 1971. Formation of teriofauna of the North Urals in the late Anthropogene. *Trudy Zoologicheskogo Instituta SSSR* 49:44–122.

Losey, R. J., L. S. Fleming, T. Nomokonova, A. V. Gusev, N. V. Fedorova, S. Garvie-Lok, O. P. Bachura, P. A. Kosintsev, and M. V. Sablin. 2018. Human and dog consumption of fish on the lower Ob river of Siberia: Evidence for a major freshwater reservoir effect at the Ust’-Polui site. *Radiocarbon* 60 (1):239–260.

Moshinskai͡a, V. I. 1953. Material'nai͡a kul'tura i khozi͡aĭstvo Ust'-Polui͡a. Pages 72-106 *in* V. N. Chernet͡sov, V. I. Moshinskai͡a, and I. A. Talit͡skai͡a, editors. *Drevni͡ai͡a istorii͡a Nizhnego Priob'i͡a* . Izd. Akademii nauk, Moscow, Russia.

Nomokonova, T., R. J. Losey, A. V. Plekhanov, and H. J. McIntyre. 2018. Iarte VI and Late Holocene reindeer remains from the Iamal Peninsula of Arctic Siberia. *Arctic Anthropology* 55 (in press).

Shii͡atov, S. G., P. M. Khantemirov, V. M. Gori͡achev, L. I. Agafonov, and M. A. Gurskai͡a, 2005. Dendrokhronologicheskie datirovki arkheologicheskikh, istoricheskikh i ėtnograficheskikh pami͡atnikov Zapadnoĭ Sibiri. Pages 43-57 *in* E. N. Chernykh, and V. I. Zav'i͡alov, editors. *Arkheologii͡a i estestvennonauchnye metody. Sb. Stateĭ* . I͡Azyki slavi͡anskoĭ kul'tury, Moscow, Russia.

Shii͡atov, S. G., and R. M. Khantemirov. 2000. Dendrokhronilogicheska datirovka drevesiny kustarnikov iz arkheologicheskogo poselenii͡a I͡Arte 6 na polustrove I͡Amal. Pages 112-120 *in* A. V. Golovnëv, editor. *Drevnosti I͡Amala*. YrO RAN, Ekaterinburg – Salekhard, Russia.
